# Revisiting 30 years of biofunctionalization and surface chemistry of inorganic nanoparticles for nanomedicine

**DOI:** 10.3389/fchem.2014.00048

**Published:** 2014-07-15

**Authors:** João Conde, Jorge T. Dias, Valeria Grazú, Maria Moros, Pedro V. Baptista, Jesus M. de la Fuente

**Affiliations:** ^1^Harvard-MIT Division for Health Sciences and Technology, Institute for Medical Engineering and Science, Massachusetts Institute of TechnologyCambridge, MA, USA; ^2^Nanotherapy and Nanodiagnostics Group, Instituto de Nanociencia de Aragon, Universidad de ZaragozaZaragoza, Spain; ^3^CIGMH, Departamento de Ciências da Vida, Faculdade de Ciências e Tecnologia, Universidade Nova de LisboaCaparica, Portugal; ^4^Fundacion ARAIDZaragoza, Spain; ^5^Key Laboratory for Thin Film and Microfabrication Technology of the Ministry of Education, Department of Bio-Nano Science and Engineering, Institute of Nano Biomedicine and Engineering, Research Institute of Translation Medicine, Shanghai Jiao Tong UniversityShanghai, China

**Keywords:** biofunctionalization, chemistry surface, gold nanoparticles, magnetic nanoparticles, quantum dots

## Abstract

In the last 30 years we have assisted to a massive advance of nanomaterials in material science. Nanomaterials and structures, in addition to their small size, have properties that differ from those of larger bulk materials, making them ideal for a host of novel applications. The spread of nanotechnology in the last years has been due to the improvement of synthesis and characterization methods on the nanoscale, a field rich in new physical phenomena and synthetic opportunities. In fact, the development of functional nanoparticles has progressed exponentially over the past two decades. This work aims to extensively review 30 years of different strategies of surface modification and functionalization of noble metal (gold) nanoparticles, magnetic nanocrystals and semiconductor nanoparticles, such as quantum dots. The aim of this review is not only to provide in-depth insights into the different biofunctionalization and characterization methods, but also to give an overview of possibilities and limitations of the available nanoparticles.

## Introduction

Every object with at least one characteristic dimension between 1 and 100 nm can be defined as “nanomaterial.” The importance of nanomaterials (e.g. nanoparticles, NPs) for science and technology has highly increased in the last years (Surendiran et al., 2009). When dealing with such a small structure, the size-related properties, the shape and inter-particle distance to the core, the charge, the dielectric properties of the conjugated system (including refractive index and polarizability), the dielectric medium surrounding the particle (solvent) and the composition moieties are extremely important and may sturdily influence the physical and chemical characteristics of the nanomaterials. This means that these distinct properties, such as quantum confinement in semiconductor nanocrystals or surface plasmon resonance (SPR) in some metal NPs, may influence physical and chemical behavior of nanomaterials (Bellucci, 2009; Doria et al., 2012).

The unbelievable development of nanotechnology in the last 30 years has allowed the release of new and efficient synthetic routes toward the production and functionalization of different NPs, composed of a variety of materials including noble metals [e.g. gold and silver (Conde et al., 2012c; Doria et al., 2012; Dreaden et al., 2012)], semiconductors [e.g. CdSe and CdTe (Murray et al., 1993), TiO_2_ (Sudhagar et al., 2012), InP (Xu et al., 2006)], magnetic compounds (Pankhurst et al., 2003), and their combinations, such as core–shell (Cao et al., 2001) and alloy NPs (Doria et al., 2010).

The unique characteristics of these NPs, such as high surface-to-volume ratio or size-dependent optical and magnetic properties, are drastically different from those of their bulk materials and hold pledge in the clinical field (Kim, 2007; Heath and Davis, 2008). Technological advances in nanoparticle synthesis/functionalization are producing significant advances in molecular detection and imaging, target and multifunctional therapeutics and in prevention/control of diseases. Through the development of new imaging agents, novel multifunctional targeted devices capable of overcoming biological barriers for direct delivery of therapeutic agents to diseased cells and tissues, and innovative monitoring sensors for predictive molecular changes, increase the processes' efficiency while minimizing costs (Baptista, 2009; Minelli et al., 2010; Ma et al., 2011; Conde et al., 2012c). Besides being an area of intense upfront basic research, nanotechnology holds the key to future technological applications. It is a burgeoning field as more and improved techniques are becoming available for clinical therapy and diagnostics with increased sensitivity and efficiency at lower costs (Baptista et al., 2008; Lammers et al., 2011).

In spite of these advantages, NPs do have limitations. For example, their small size and large surface area can lead to particle-particle aggregation and may result in limited loading of functional components and burst release. In fact, only NPs with the appropriate size and surface chemistry are not immediately recognized by the immune system and show increased circulation times (Gil and Parak, 2008; Shvedova et al., 2010). Nevertheless, their unique and broad-based optical properties, their ease of synthesis and facile surface chemistry and, most importantly, their appropriate size scale are overcoming these drawbacks and have been generating much eagerness in clinical diagnostics and therapy (Sperling et al., 2008).

Here we will review different strategies of biofunctionalization and characterization methods of inorganic nanoparticles, as well as their main advantages and limitations. Our aim is to discuss the challenges of working with nanoparticles while giving an overall overview of the state-of-the-art.

## Inorganic nanoparticles

### Gold nanoparticles

Historically, colloidal gold has been used since ancient times mainly as a method for glass staining. However, it was not until Faraday's work that gold nanoparticles (AuNPs) began to be studied in a scientific approach (Faraday, 1857). Since then, AuNPs have generated ever-increasing interest and in the last few decades more and more controllable synthesis methods and applications in diverse nanosystems have been developed (Wagner et al., 2000; Edwards and Thomas, 2007).

AuNPs, also known as colloidal gold, are a suspension of sub-micrometer-sized gold metal particle in a fluid and can be obtained with diameters between 3 and 200 nm. AuNPs have gained increasing interest due to their special features, such as extraordinary optical and electronic properties, high stability and biological compatibility, controllable morphology and size dispersion, and easy surface functionalization (Sperling et al., 2008).

The optical properties of AuNPs are significant because absorption and emission are within the visible range of light (El-Sayed, 2001). Of particular interest is the light extinction process in the UV-visible range, which occurs when an electromagnetic wave passes through a metal particle exciting its electronic or vibrational states (Kreibig and Vollmer, 1995). This phenomenon induces dipole moments that oscillate at the respective frequency of the incident wave, dispersing secondary radiation in all directions. This collective oscillation of the free conduction electrons is called localized surface plasmon resonance (LSPR). The SPR is the collective oscillation of the electrons in the conduction band. The oscillation frequency is usually in the visible region giving rise to the strong SPR absorption (Schultz et al., 2000; Jain et al., 2006; Huang et al., 2007; Murphy et al., 2008).

Size also provides important control over many of the physical and chemical properties, including luminescence, conductivity, and catalytic activity. AuNPs' absorption and scattering proportions depend on the AuNPs size (Cao et al., 2002). AuNPs with a diameter smaller than 20 nm essentially show absorption, but when the size increase to 80 nm the ratio of scattering to absorption also increases. As the size of the AuNPs increases, light can no longer polarize the nanoparticles homogeneously and higher order modes at lower energy dominate. This causes a red-shift and broadening of the surface plasmon band. Small AuNPs, like those with 13 nm of diameter, absorb green light, which corresponds to a strong absorption band at 520 nm in the visible light spectrum. However, solutions of AuNPs appear red in color. For smaller AuNPs (i.e., 5 nm diameter), surface electrons are oscillated by the incoming light in a dipole mode, but the SPR is very sensitive to the composition, size, shape, inter-particle distance and environment (dielectric properties) of the AuNPs (Pellegrino et al., 2005; Sperling et al., 2006).

In all synthesis methods reported so far, a reducing and a stabilizing agent is added to prevent the particles from aggregating. The type of stabilizer affects the selection of further biofunctionalization strategies, as for certain strategies a good colloidal stability at a broad range of pH and ionic strength is required. Although a wide variety of nanoparticles can be stabilized by a large range of stabilizers (ligands, surfactants, polymers, dendrimers, biomolecules) (Sperling and Parak, 2010), the most robust nanoparticles are covered by thiol molecules using the strong gold–S bond between the soft acid Au and the soft thiolate base (Giersig and Mulvaney, 1993). Thus, if a bifunctional thiolated stabilizer or even a mixture of them is used (e.g. thiolated-PEG molecules having carboxylic, amine, azide groups, etc.), then it is possible to stabilize and introduce chemical functionalities in a single step. This allows further attachment of biomolecules in a controlled way by covalent immobilization strategies that we will discuss in the following sections.

### Magnetic nanoparticles

Magnetic nanoparticles (MNPs) constitute an important class of nanomaterials widely studied for their potential use in biomedicine fields, such as imaging, cell labeling, hyperthermia and drug and gene delivery (Pankhurst et al., 2003; Barakat, 2009; Berry, 2009). MNPs can be classified as metal, alloys or oxides, and are generally based on elements such as iron, cobalt, nickel, or manganese among others (Pankhurst et al., 2009). From the aforementioned, iron-based NPs are the most studied, since iron is believed to be biocompatible (Hanini et al., 2011). Other elements, such as cobalt or nickel are reported to be more toxic (Fang and Zhang, 2009). Iron oxide NPs (IONPs) are composed of magnetite (Fe_3_O_4_) or maghemite (γ-Fe_2_O_3_), nanocrystallites.

Most of MNPs' applications are a consequence of their magnetic properties, which greatly differ from those of the bulk material. When small enough, MNPs present superparamagnetic behavior at room temperature, meaning that they become magnetized upon exposure to an external magnetic field but lack remnant magnetization once the external field is removed (Jun et al., 2008). Consequently, MNPs do not agglomerate in the absence of the magnetic field, which is essential for *in vivo* applications (Yoo et al., 2011). This characteristic is rather important in applications, such as magnetic hyperthermia. The MNPs' capacity of converting the energy of an alternate magnetic field into heat (Rosensweig, 2002) and the extra sensitiveness of tumor cells to an increase in temperature (van der Zee, 2002) are the two pillars of magnetic hyperthermia in cancer. Since the late 50's, when Gilchrist et al. (1957) first reported the use of MNPs to heat tissue samples, to nowadays, magnetic hyperthermia has evolved considerably and is a key area of interest in cancer therapy with several studies showing the benefit of employing magnetic materials in hyperthermia strategies (Jordan et al., 1993, 2001; Johannsen et al., 2010; Laurent et al., 2011). Several groups have reported noteworthy results in clinical trials where magnetic hyperthermia shows effectiveness in tumor cell destruction with impressive targeting, thus minimizing significantly side effects (Johannsen et al., 2005; Liu et al., 2011; Zhao et al., 2012b).

There are a wide variety of methodologies used for MNP synthesis, including physical or wet chemical approaches. Concerning wet chemical approaches, there are some methodologies, such as coprecipitation (Perez et al., 2002) or reverse micelles precipitation (Liu et al., 2000) that provide directly water soluble MNPs with an organic layer with chemical moieties for narrow size distribution of MNP. However, common synthetic strategies traditionally render MNPs soluble only in organic solvents. Their use in bioapplications imply an additional step where adequate chemical moieties are introduced by several strategies (e.g. use of amphiphilic polymers, silanization, replacing and/or modifying the surfactant layer) in order to allow silanization, their water transference and further biofunctionalization.

### Quantum dots

Quantum dots (QDs) are nanoparticles composed of semiconductor materials from III-V or II-VI groups of the periodic table, such as ZnS, ZnSe, CdS, CdSe, CdTe, InP, and others (Donega, 2011). Their reduced size induces a shift of the electronic excitations to higher energy, concentrating the oscillator strength into just a few transitions, conferring unique quantum-confined photonic and electronic properties (Alivisatos, 1996; Alivisatos et al., 2005). Although physically larger than organic dyes and fluorescent proteins, their cumulative optical properties offer great biological utility. With tunable core sizes, it is possible to attain a broad adsorption profile, narrow size, and symmetric photoluminescence spectra depending of the fundamental materials. QDs also show strong resistance to photobleaching and chemical degradation, as well as significant photostability and high quantum yields (Ghanem et al., 2004; Xu et al., 2006; Algar et al., 2011).

Their potential as biological labels was first demonstrated by Nie and Alivisatos groups in 1998, turning the focus into bioapplications of QDs. The method relies on a ligand exchange strategy is based on the replacement of the original hydrophobic ligands adsorbed onto the surface of QDs with biofunctional molecules, such as protein transferrins. These QDs were susceptible to effective receptor-mediated endocytosis in cultured HeLa cells. Since these first demonstrations of QDs potential, their unique properties have been continuously optimized and applied in a plethora of bioapplications, ranging from fluorescent probes, biosensors to therapeutics and theranostic agents (Akerman et al., 2002; Smith et al., 2006; Li et al., 2009; Liu et al., 2010; Ruan et al., 2012; Singh et al., 2012).

Once QDs that show paramount optical properties are those synthesized in organic media, numerous methods have been developed for creating hydrophilic QDs (Medintz et al., 2008). The first approach is commonly designated as “ligand exchange” (Gill et al., 2008), where the hydrophobic layer of the organic solvent may be replaced by biofunctional molecules containing a soft acidic group (i.e., thiol, sodium thiolycolate) and hydrophilic groups (i.e., carboxylic, aminic groups) (Wang et al., 2008). A second approach usually consists in adding a particular shell to the nanoparticles that can be further functionalized with additional biomolecules or polymers (Koole et al., 2008; Zhang et al., 2008).

## Biofunctionalization of inorganic nanoparticles

**Figure 1:**
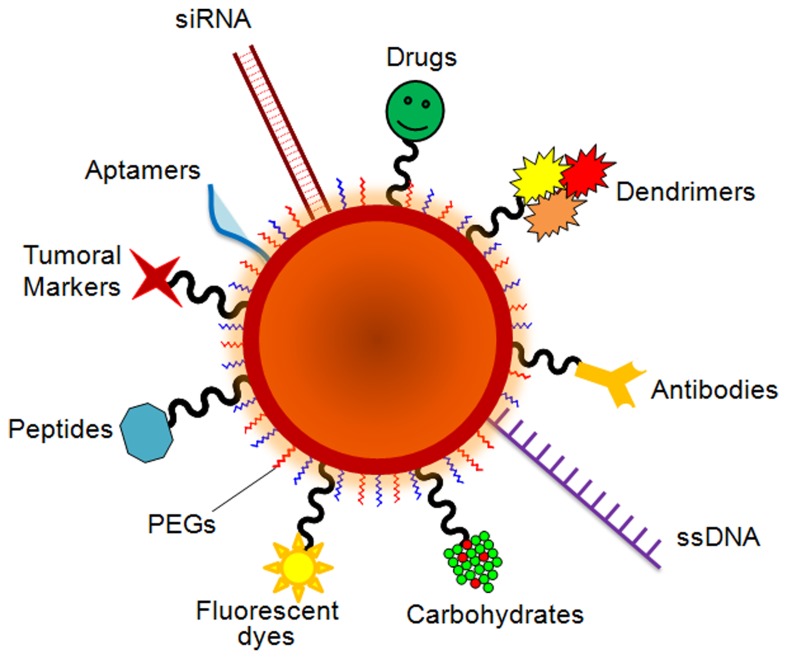
**Schematic representation of a multifunctional nanocarrier**. These innovative NPs comprise nucleic acids such as RNA and DNA used for gene silencing approaches and in colorimetric assays, respectively. Aptamers and anticancer drug molecules are also used for delivery to the target tissue. Carbohydrates may be useful as sensitive colorimetric probes. PEG is used to improve solubility and decrease immunogenicity. Responsive nanocarriers can also trigger reaction upon external stimuli through the functionality of valuable tumor markers, peptides, carbohydrates, polymers and antibodies that can be used to improve nanocarrier circulation, effectiveness, and selectivity. Multifunctional systems can also carry fluorescent dyes that are used as reporter molecules tethered to the particle surface and employed as tracking and/or contrast agents.

Nanoparticles with unique and broad-based optical properties, ease of synthesis and facile surface chemistry and functionalization within appropriate size scale are generating much enthusiasm in biotechnology and biomedicine, with particular emphasis in clinical diagnostics and therapy. However, for the biological application of these NPs, it is necessary their functionalization with one or several biomolecule (Figure 1), such as DNA/RNA, oligonucleotides (i.e., ssDNA/RNA, dsDNA/RNA), peptides and antibodies, fluorescent dyes, polymers (i.e., PEGs), drugs, tumor markers, enzymes and other proteins that will introduce the required bio-functionalities.

Ultimately, the conjugation strategy is directly dependent on a numbers of factors such as size, surface chemistry and shape, as well as the type of ligands and functional groups to exploit in the functionalization. Also, the type of biological molecule and the final application of the nanoparticle conjugate are crucial when evaluating the conjugation strategy. Next, we summarize the most frequently used biofunctional molecules used to introduce one or several biological activities to the NP.

### Common biofunctional species

#### Polymer coatings—poly(ethylene glycol)

For their use as potential delivery devices *in vivo*, the aforementioned inorganic nanoparticles must have long plasma half-lives. In this sense, poly(ethylene glycol) (PEG) is the most widely used macromolecule to prolong nanocarriers half-life. In fact, PEGs have a strong effect on nanoparticle structure, stabilization and biodistribution both *in vitro* and *in vivo* (Akerman et al., 2002; Daou et al., 2009; Boeneman et al., 2010; Maldiney et al., 2011). These long-circulating nanoparticles have the ability to circulate for a prolonged period of time and target a particular organ, as carriers of DNA in gene therapy, or to deliver proteins, peptides and drugs (Langer, 2000; Bhadra et al., 2002; Kommareddy et al., 2005; Lee and Kim, 2005).

For systemic applications, the development of surface functionalized and long-circulating NPs as cellular probes and delivery agents is highly desired for passive targeting to tumors and inflammatory sites. PEG-modification of NPs affords long circulating property by evading macrophage-mediated uptake and removal from the systemic circulation. Owing to its simple structure and chemical stability, it is a prototype of an inert and biocompatible polymer (Sperling and Parak, 2010; Verma and Stellacci, 2010). When bound to surfaces, PEG prevents other molecules to bind by steric effects. In fact, the molecules are not attracted by electrostatic forces and cannot penetrate the hydrated PEG layer, producing an inert hydrophilic surface. Moreover, PEG modified nanoparticles are more stable at high salt concentrations and in biological environments, avoiding non-specific binding to proteins and cells (Sperling and Parak, 2010). This is particularly important for *in vivo* applications because once the NPs are in the bloodstream, a portion of the plasma proteins that can adsorb to the surface (opsonins), may promote NPs recognition by the mononuclear phagocyte system (MPS), and consequently lead to rapid removal of the NPs from circulation (Bertrand and Leroux, 2012). To date, there is a general consensus that to prolong NPs half-life in the organism, PEGs' molecular weight, grafting density and chain architecture must be optimized (Li and Huang, 2010; Grazú et al., 2012). For instance, Xie and coworkers showed that MNPs functionalized with PEG with molecular weights higher than 3000 Da were not taken up by macrophages *in vitro*, while extensive uptake was observed for PEG 600-coated MNPs (Xie et al., 2007).

**Figure 2:**
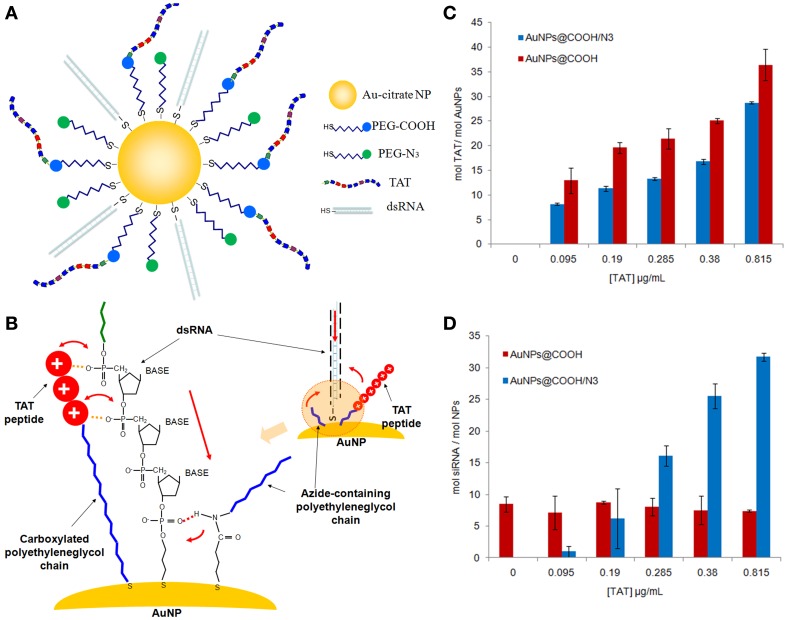
**Polyvalent PEGylated gold nanoparticles. (A)** Bioconjugation of the surface-modified gold nanoparticles with different thiol-PEG layer composition (SH-EG(7)-CH_2_-COOH and SH-(CH_2_)_3_-CONH-EG(6)-CH_2_-N_3_), TAT peptide and thiol-dsRNA oligonucleotide. **(B)** Mechanism for the enhancement of the dsRNA loading on AuNPs functionalized with PEG chains and TAT peptide. The azide group has a resonant structure with a positively polarized behavior that can attach the negatively charged thiolated oligonucleotide to the gold surface. The azide-containing chain also encloses an amide group near the gold surface that could play a role in approaching the thiol group of the oligonucleotide to the gold surface. This amide group could form a hydrogen bond with one of the hydroxyl groups of the ribose group near the thiol group on the oligonucleotide. **(C)** Determination of the number of TAT chains bound to AuNPs by the EDC reaction as a function of the initial peptide concentration in the reaction mixture. Blue bars AuNP@COOH/N_3_ and red bars AuNP@COOH. **(D)** Loading of thiolated oligonucleotide (HS-dsRNA) on AuNPs functionalized with TAT peptide and with both PEG-azide and PEG-COOH and only with PEG-COOH. Blue bars AuNP@COOH/N_3_ and red bars AuNP@COOH (Sanz et al., 2012). Reproduced with permission from Sanz et al. (2012), Copyright 2013.

Consequently, functionalization of NPs with a high density of PEG of an adequate length not only increases the colloidal stability of the modified NPs but also their plasma half-life. However, to provide PEGylated NPs with targeting and therapeutic activity, as well as with the ability of crossing different biological membranes, they must be conjugated with a variety of biologically relevant ligands, such as cell/tumor penetrating peptides, tumor markers, and therapeutic agents (siRNAs, drugs). Concerning gold NPs, one of the main strategies is to assemble PEG and mixed biomolecule/PEG monolayers on the nanoparticles' surface. Liu et al. showed an escalation in the NPs' stability with increasing PEG length, decreasing nanoparticle diameter, increasing PEG mole fraction and mixed monolayers prepared via the sequential addition of PEG followed by a peptide. In this manner, NPs were more stable than those prepared via simultaneous co-adsorption. These modified NPs were able to target the cytoplasm of HeLa cells, being the cellular uptake quantified using inductively coupled plasma optical emission spectrometry (Liu et al., 2007). Sanz et al. also obtained polyvalent PEGylated AuNPs with a similar strategy. The authors developed an approach to attach specific biomolecules to the AuNPs' surface and their effect in the functionalization with other specific derivatives. The effect of biofunctional spacers, such as thiolated PEG chains and a positive peptide (TAT) in dsRNA loading on AuNPs was reported. The authors hypothesized that the loading of oligonucleotides onto the AuNP surface may be controlled by ionic and weak interactions positioning the entry of the oligonucleotide through the PEG layer, by a synergistic effect of the TAT peptide and PEG chains with specific functional groups, enhancing the dsRNA loading onto AuNPs (see Figure 2) (Sanz et al., 2012).

Another approach to link biomolecules to PEGylated AuNPs is making use of PEG as a spacer. This requires the use of bifunctional PEG chains that contain thiol at one end and a suitable functional moiety at the other (e.g. amino, carboxylate groups). Recently, Oh et al. described a different approach, where in a one-phase synthesis AuNPs were conjugated with PEG ligands yielding a narrow size distribution of highly stable NPs in the presence of high salt concentrations over a wide range of pHs (Oh et al., 2010b). One way or another, functional moieties of PEG ligands allow for further coupling of target biomolecules. Consequently, surface modification of gold clusters through PEG spacers (Kanaras et al., 2002; Simpson et al., 2011) would allow the modified nanoparticles to remain in the systemic circulation for prolonged periods and provide flexibility for efficient interaction with a target. Besides, using a combination of different bifunctional PEG spacers, gold nano-platforms can be multifunctionalized with a variety of biologically-relevant ligands such as cell penetrating peptides, fluorescent dyes, tumor markers and siRNA (Conde et al., 2012a).

PEGylated QDs have also been successfully produced for effective *in vitro* and *in vivo* circulation (Skaff and Emrick, 2003; Hu et al., 2010; Prow et al., 2012; Yang et al., 2012b). Recently, Poulose et al. developed highly biocompatible PEG functionalized in cadmium chalcogenide luminescent QDs (CdS, CdSe, and CdTe) as an imaging tool for early diagnosis of cancer by targeting a cancer cell line (Poulose et al., 2012).

Although PEG is really useful to prolong NPs' blood half-life, it is known that in some cases PEG can hamper cargo release or hide other functional domains once the NP accumulates at the desired target area (Sawant and Torchilin, 2012). Inclusion of stimulus-sensitive detachable PEG is a possible solution to overcome these drawbacks, so that cargoes can be released or other ligands unveiled in response to microenvironmental conditions. For instance, Harris et al. functionalized MNPs with a PEG tethered by an MMP-2 cleavable substrate (Harris et al., 2008), being MMP-2 a protease upregulated in angiogenesis and metastasis. Once NPs reached the tumor, the polymer was cleaved, unveiling the cell penetrating peptide, resulting in increased uptake by cells when compared to non-cleavable PEG.

#### Fluorescent dyes

Several studies report on the modulation of fluorophores at the vicinity of nanoparticles (Kang et al., 2011; Rosa et al., 2011), which has found application in a variety of systems to detect biologically relevant targets.

**Figure 3:**
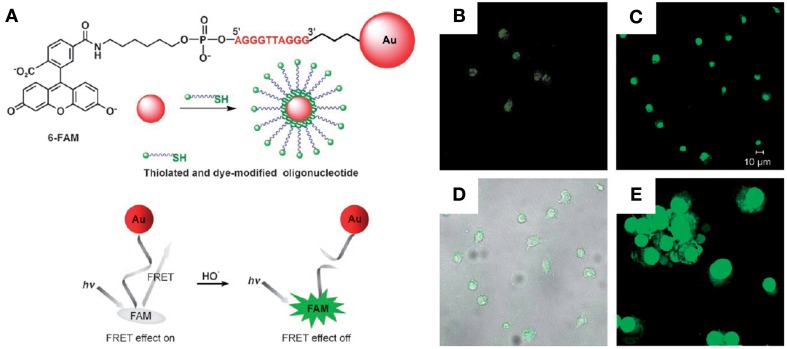
**Fluorescent-AuNPs. (A)** Chemical structure of a FAM–DNA–AuNP and schematic illustration of its FRET-based operating principles. **(B–E)** Confocal fluorescence and phase-contrast images of living cells. **(B)** Fluorescence image of macrophages incubated with the probe for 30 min at 37°C. **(C)** Fluorescence image of probe-stained macrophages stimulated with PMA for 1 h at 37°C. **(D)** Bright field image of live macrophages shown in **(C)**, confirming their viability. **(E)** AO staining of probe-loaded macrophages, confirming their viability (Tang et al., 2008). Reproduced with permission from Tang et al. (2008), Copyright 2013.

Several methods based on the quenching of fluorescence have been proposed for DNA detection consisting of fluorophore-labeled ssDNA electrostatically adsorbed onto AuNPs (Ray et al., 2006), where the presence of a complementary target triggers desorption of the newly formed dsDNA from the nanostructures due to the electrostatic variation between ssDNA and dsDNA, and fluorescence emission is restored. Also, fluorescence quenching of fluorophores close to gold nanocarriers functionalized with thiol-modified oligonucleotides has been explored in different conformations (Wu et al., 2006; Tang et al., 2008). Tang et al. proposed a method to probe hydroxyl radicals using an AuNP-oligonucleotide-FAM system where the hydroxyl radical promotes strand breakage and consequent release of FAM, restoring the previously quenched fluorescence (see Figure 3) (Tang et al., 2008). The same quenching mechanism was used to detect specific DNA strands using two probes (one with an AuNP label and another labeled with TAMRA) that hybridize to two DNA sequences near each other (Wu et al., 2006), bringing the fluorophore and AuNP close enough to quench fluorescence emission.

Proteins have also been probed through nanoparticle fluorescence-mediated systems, especially for protein detection via quenching, through the interaction with fluorescent AuNPs (Mayilo et al., 2009; He et al., 2010; Lacerda et al., 2010). Due to their efficient proximity-dependent fluorescence quenching they can be used *per se* or as part of more elaborate conjugates (i.e., with QDs) (Pons et al., 2007). One example is the work of De et al. where the interaction between AuNPs and the green fluorescent protein (GFP) was employed to detect proteins in biological matrices, such as serum (De et al., 2009). By correlating the variation in fluorescence intensity with specific proteins of interest, they were able to identify proteins such as fibrinogen, human serum albumin and immunoglobin G, among others with over 97% accuracy.

In essence, fluorescent-nanoparticle systems can be used for sensing by exploring a typical FRET in order to provide efficient *in vivo* detection and tumor targeting. These nanocarriers symbolize an important class of materials with unique features suitable for biomedical imaging applications such as increased sensitivity in detection, high quantum yields for fluorescence and a bounty of novel applications in optics and nanophotonics for molecular diagnostics (Conde et al., 2012d).

QDs are often used as fluorescent molecules *per se*, since they are semiconductor nanoparticles with narrow, tunable, symmetrical emission spectra and high quantum yields (Weller, 1993; Bruchez et al., 1998). These characteristics were evidenced by Wu et al. using QDs modified with different cellular antigens enabling the simultaneous detection of two different targets in the same cell (Wu et al., 2003). It was also shown their higher brightness and photobleaching resistance when compared to organic dyes. These properties make QDs exceptional substitutes as fluorescence labels (Xing et al., 2006; Smith et al., 2010; Smith and Nie, 2012).

The inclusion of dyes onto MNPs allows the creation of multifunctional NPs, which might be used for MRI and optical imaging. These dual MNPs allow for multimodal imaging, which implies that the limitations of one imaging modality could be compensated by the other, creating a complementary effect (Louie, 2010). For instance, Medarova and co-workers reported the synthesis of a multifunctional MNP that included near-infrared optical imaging dye, peptides for membrane translocation and synthetic siRNA targeting a specific gene (Medarova et al., 2007). *In vivo* accumulation of the MNPs was assessed by MRI and optical imaging and the silencing efficiency was also probed by *in vivo* optical imaging.

#### Nucleic acids

Watson and Crick first described DNA as two helical chains each coiled around the same axis, consisting of simple and repeating units called nucleotides with backbones made of sugars and phosphate groups joined by ester bonds that run in opposite directions to each other. The importance of this molecule within living cells is undisputable (Watson and Crick, 1953). Besides their biological function, nucleic acids can be employed as polymeric molecules which will bind specifically to targets thanks to Watson–Crick base pairing (Fichou and Ferec, 2006).

Mirkin et al. (1996) described the use of a cross-linking method that relies on the detection of single-stranded oligonucleotide targets using two different gold nanoprobes, each of them functionalized with a DNA-oligonucleotide complementary to one half of the given target. This functionalization was achieved using the strong affinity of thiol or disulfide groups to the gold surface of the NPs, forming quasi-covalent bonds. By modifying a nucleic acid molecule with a thiol group in either the 5′ or the 3′ end it is possible to fine-tune the DNA assembly into the gold surface (Hurst et al., 2006), controlling variables such as salt concentration, oligo/NP ratio or nanoparticle size. This phenomenon indicates the potential of AuNPs modified with DNAs to be applied in biosensing or as DNA probes for diagnosis (Cao et al., 2005). These assays became an important mark in detection once they have PCR-like sensitivity, selectivity for target sequences, capacity for massive multiplexing, and most importantly, have the ability to be performed at the point of care.

**Figure 4:**
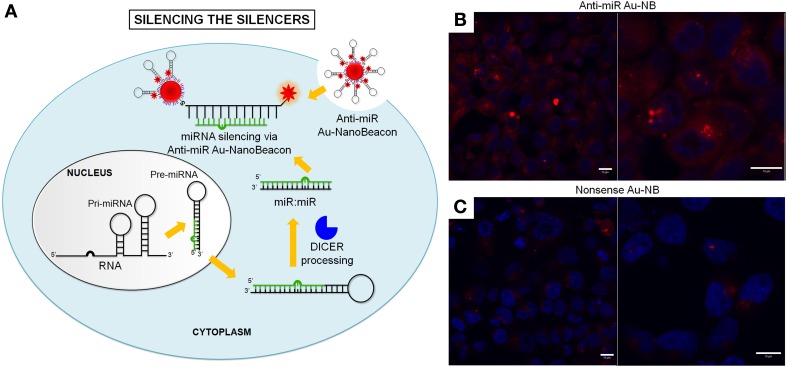
**Silencing the Silencers with hairpin-DNA-AuNPs—Gold nanobeacons. (A)** Specific Au-nanobeacons are capable of intersecting miRNA pathway, leading to recovery of previously downregulated gene expression while simultaneously discriminating cells where silencing is occurring. The fluorescence signal may allow for tracking cell internalization and sub-cellular localization. The Au-nanobeacons' potential for anti-cancer therapeutics via the silencing of the silencers is demonstrated by blocking the endogenous microRNA pathway via an Anti-miR Au-nanobeacon complementary to the mature microRNA-21 (miR-21), commonly upregulated in cancer phenotypes. **(B,C)** Au-nanobeacons silencing of endogenous silencers—silencing of miR-21. Confocal imaging (scale bar, 10 μm) shows internalization of 50 nM **(B)** Anti-miR Au-nanobeacon 50 nM and **(C)** Nonsense Au-nanobeacon. Target (mature miR-21) recognition leads to change of Anti-miR Au-nanobeacon conformation in the cytoplasm with concomitant fluorescence signal (red, Cy3) encircling the cell nuclei (blue, DAPI) (Conde et al., 2013b). Reproduced with permission from Conde et al. (2013b), Copyright 2013.

Using a fluorescence-based method, Demers et al., have determined the number of thiol-derivatized single-stranded oligonucleotides bound to AuNPs and their extent of hybridization with complementary oligonucleotides in solution (Demers et al., 2000). Also, using a fluorescence method, Conde et al. reported the potential of a single molecular nanoconjugate to intersect all RNA pathways: from gene specific downregulation to silencing the silencers, i.e., siRNA and miRNA pathways, by using gold nanobeacons (Figure 4). These nanoconjugates functionalized with a fluorophore labeled hairpin-DNA are capable of efficiently silencing single gene expression, exogenous siRNA and an endogenous miRNA while yielding a quantifiable fluorescence signal directly proportional to the level of silencing. Inhibition of gene expression was achieved with concomitant increase of the gold nanobeacons' fluorescence that can be used to assess the silencing effect (Rosa et al., 2012; Conde et al., 2013a,b, 2014b).

Moreover, AuNPs functionalized with ssDNA are also capable of specifically hybridizing to a complementary target for the detection of a particular nucleic acid sequence in biological samples (Sato et al., 2003; Storhoff et al., 2004; Baptista et al., 2005; Thaxton et al., 2006). The impact of advances in these nanoparticle-based assays for specific detection of bioanalytes of clinical interest is particularly relevant in biodiagnostics, making them ideal candidates for developing biomarker platforms.

However, nucleic acid molecules are also capable of establishing ionic interaction in gold surface. It has been shown that both thiol-ssDNA and dsDNA stabilize gold nanoparticle dispersions, but possible non-specific interactions between the hydrophobic DNA bases and the gold surface promote interparticle interactions and cause aggregation within a short period of time (Cardenas et al., 2006). The charge repulsion among DNA strands and between DNA and AuNPs can be reduced by adding salt, reducing pH or by using non-charged peptide nucleic acid (PNA) (Zhang et al., 2012b).

**Figure 5:**
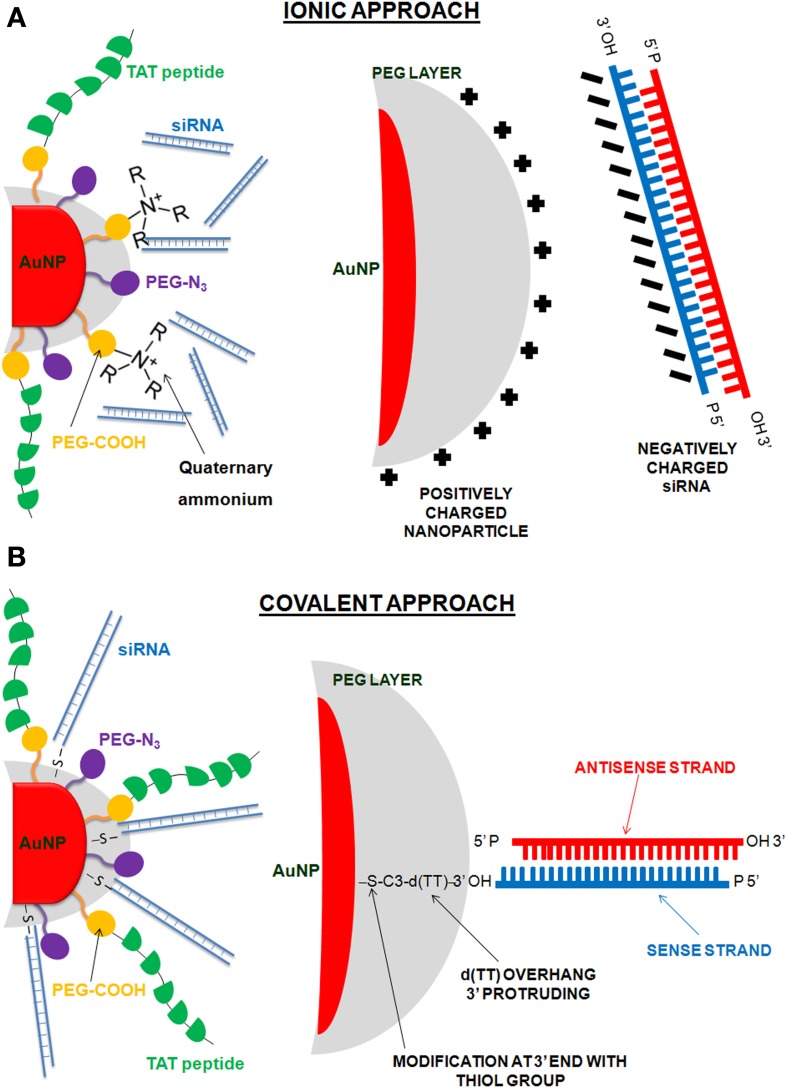
**Gold nanoparticles functionalized with multiple biomolecules: PEG, cell penetration peptide (TAT), ammonium quaternary groups, and siRNA**. Two different approaches were employed to conjugate the siRNA to the AuNPs: **(A)** ionic approach, interaction of the negatively charged siRNA to the modified surface of the AuNPs through ionic interactions; **(B)** covalent approach, use of thiolated siRNA for gold thiol binding to the NPs.

Moreover, Conde et al. reported the design of two approaches (see Figure 5) for the binding of siRNA molecules to multifunctional AuNPs: (Figure 5A) the binding of the negatively charged siRNA through ionic interactions to the modified gold surface (ionic approach) and (Figure 5B) the use of thiolated siRNA for the binding to the nanoparticle through the strong interaction gold-thiol (covalent approach) (Conde et al., 2012a). The covalent approach was evaluated in a lung cancer mouse model to evaluate the inflammatory response and therapeutic siRNA silencing via RGD-nanoparticles. This study reported the use of siRNA/RGD gold nanoparticles capable of targeting tumor cells in two lung cancer xenograft mouse models, resulting in successful and significant *c-Myc* oncogene downregulation followed by tumor growth inhibition and prolonged survival of the animals (Conde et al., 2013c).

Modulation of the physicochemical properties of the gold nanocarriers can be easily achieved by adequate synthetic strategies and give AuNPs advantages over conventional detection methods currently used in clinical diagnostics and therapy. Simple and inexpensive methods based on these bio-nanoprobes were initially applied for detection of specific DNA sequences and are currently being expanded to clinical diagnosis (Baptista et al., 2008).

QDs have also been functionalized with nucleic acids and extensively used as DNA sensors (Crut et al., 2005; Dubertret, 2005; Zhang et al., 2005a; Choi et al., 2009; Ye et al., 2009; Zhang and Hu, 2010). For *in vitro* test of target DNA, QDs based FRET pairs turned out to be of great use. The method developed by Zhang et al. using QDs-Cy5-labeled reporter oligonucleotide conjugates, capable of detecting low concentrations of DNA in a separation-free format. This system uses QDs linked to DNA probes to capture DNA targets and successfully detect a point mutation (Zhang et al., 2005a).

Owing to their unique optical properties, QDs have also been applied for multiplex detection of analytes with single-molecule detection. Zhang et al. reported the use of a single QD-based nanosensor for multiplex detection of HIV-1 and HIV-2 at single-molecule level in a homogeneous format (Zhang and Hu, 2010). These QD-based nanosensors have several advantages such as extremely low sample consumption, high sensitivity, short analysis time and have the potential to be applied for rapid point-of-care testing, gene expression studies, high-throughput screening, and clinical diagnostics.

When modified with DNA, QDs were successfully employed in the detection of respective complementary DNA strands via FRET. Sub-nanomolar detection limits have been reported (Zhou et al., 2008a; Singh and Strouse, 2010). The strategy success is directly related to the covalent coupling of the nucleic acid molecule to the QD, controlling the donor-acceptor distance, fundamental in FRET-based biosensors.

QDs can also be used in gene delivery. Jin-Ming Li et al. developed a series of QDs functionalized with β-cyclodextrin coupled to amino acids. Using the β-cyclodextrin as a vector for delivering doxorubicin (DOX) and electrostatically binding MDR1 siRNA, this strategy allowed for simultaneous chemotherapy and gene silencing. The authors observed that in HeLa cells it was possible to induce apoptosis due to the intracellular accumulation of Dox and also reduced levels of MDR1 gene expression. These multifunctional QDs are promising vehicles for the co-delivery of nucleic acids and chemotherapeutics, as well as for real-time tracking of treatment (Li et al., 2012).

QDs-siRNA conjugates have also been used for imaging and gene silencing approaches (Derfus et al., 2007; Tan et al., 2007; Yezhelyev et al., 2008; Zhao et al., 2010; Li et al., 2011, 2012). For example, Yezhelyev et al. developed multifunctional nanoparticles for siRNA delivery and imaging based on the use of QDs and proton-absorbing polymeric coatings (proton sponges). The authors demonstrated a dramatic improvement in gene silencing efficiency and simultaneous reduction in cellular toxicity, when compared with existing transfection agents. Additionally, QD-siRNA nanoparticles are also dual-modality optical and electron-microscopy probes, allowing real-time tracking and ultrastructural localization of QDs during delivery and transfection (Yezhelyev et al., 2008).

MNPs have also been frequently used as platforms for the delivery of DNA or siRNA, as they can be used to track their biodistribution by MRI. For instance, Kumar et al. synthesized multifunctional MNPs by attaching a near-infrared optical dye Cy5.5 and a peptide that targets the tumor specific antigen mucin-1 to cross-linked dextran coated SPIONs (Kumar et al., 2010). The delivery of the nanosystem to tumors in mice was imaged either *in vivo* or *ex vivo* by MRI and optical imaging.

On the other hand, the functionalization of plasmid DNA and siRNA to MNPs has been widely reported, as MNPs are used as tools for magnetofection, that is to say, the enhanced delivery of nucleic acids associated to MNPs using external magnetic fields (Scherer et al., 2002; Dobson, 2006; Plank et al., 2011). Using magnetofection the transfection efficiency can be highly improved when compared with transfections carried out with non-magnetic gene delivery systems in a variety of primary cells and cell lines (Mykhaylyk et al., 2007; Prijic and Sersa, 2011). Although magnetofection results are promising *in vitro*, and several studies have reported the systemic delivery of nucleic acids using MNPs *in vivo*, not many of them use an external magnetic field to enhance the accumulation of the MNPs in the targeted area (Plank et al., 2011). Thus far, the most promising application of magnetofection as an *in vivo* cancer therapy has been reported by Namiki et al. (2009). The authors formulated oleic acid-coated MNPs assembled with cationic lipid shells, and functionalized them with an appropriate siRNA sequence to knock down the epidermal growth factor receptor (EGFR) mRNA, as it is overexpressed in tumor blood vessel endothelium. After systemically injecting the complex to mice tumors, the authors found a 50% reduction in tumor mass when a magnetic field was applied compared to the control group without magnetic field.

#### Peptides

Peptides are short chains of amino acid monomers linked by amide bonds and are distinguished from proteins on the basis of size, once they only contain ~50 amino acids or less. Peptides can be found naturally or synthetically and have the potential for the stabilization and biofunctionalization of NPs. For instance Wang et al. demonstrate that multiple functional peptide stabilized AuNPs are readily obtained in a one-step surface coating procedure and that the surface functionalities can be selectively addressed on a microarray. The authors developed a straightforward route to stable AuNPs, both with single and with dual biological functionality. The particles exhibit the specific recognition properties of the biological cargo without any indication of non-specific binding or particle aggregation (Wang et al., 2005).

The stability conferred by peptide ligands usually depends on their length, hydrophobicity, and charge and in some cases resulted in further improved stability. Actually, Levy et al. designed a pentapeptide ligand, CALNN, which converts citrate stabilized AuNPs into extremely stable, water-soluble AuNPs with some chemical properties analogous to those of proteins. These peptide-capped AuNPs can be freeze-dried and stored as powders that can be subsequently redissolved to yield stable aqueous dispersions (Levy et al., 2004).

Biofunctional peptide sequences include “membrane translocation signals” like the HIV-TAT peptide sequence (de la Fuente and Berry, 2005; Berry et al., 2007; Conde et al., 2012a), which is capable of transporting nanoscale materials across cellular membranes and “nuclear locating signals” that could be used for further intracellular targeting (Nativo et al., 2008; Chithrani, 2010). In fact, de la Fuente et al. developed AuNPs functionalized with HIV-Tat peptide used to achieve cytoplasm and the cell nucleus. One of the major drawbacks of these cell penetrating peptides is that they are not cell specific and that they can remain entrapped in endosomes (Gump and Dowdy, 2007). To overcome these limitations, fusogenic peptides that are able to escape from endosomes or homing peptides capable of reaching specific tissues or cells have been developed (Li et al., 2004; Ruoslahti et al., 2010).

QDs labeled with these types of peptides have been extensively prepared using various strategies (Chen and Gerion, 2004; Cai and Chen, 2008; Curnis et al., 2008). For example, Akerman et al. show that ZnS-capped CdSe QDs coated with a lung-targeting peptide accumulate in the lungs of mice after intravenous injection, whereas two other peptides specifically direct QDs to blood or lymphatic vessels in tumors (Akerman et al., 2002). Curnis et al. also found that a cyclic Ciso DGRC peptide coupled to QDs could bind alphavbeta 3 integrin and co-localize with several antibodies in human renal cell carcinoma tissue sections, indicating that this peptide could efficiently recognize endothelial cells of angiogenic vessels (Curnis et al., 2008). Cai et al. also used a thiolated arginine-glycine-aspartic acid (RGD) peptide to conjugate to the QDs and applied the QDs peptide bioconjugates for tumor vasculature targeted imaging (Cai and Chen, 2008). However, if crossing the vascular wall is needed, these RGD peptides need to be improved. The Ruoshlati group reported a cyclic peptide iRGD that can combine the tumor-homing RGD sequence with a tissue penetration motif (Sugahara et al., 2009). Therefore, the homing sequence directs the peptide to the tumor vascular endothelium, while the tissue penetration motif, once activated by a protease, binds to a different receptor (neuropilin-1), which mediates extravasation and tissue penetration. As a proof of concept, iRGD peptide-linked iron oxide nanoworms could be detected by MRI throughout a tumor once injected *in vivo* to mice. Recently, the same group combined two different peptides with the magnetic nanoworms to image and treat mice with glioblastoma, one of the most difficult tumors to treat (Agemy et al., 2011). While the CGKRG peptide targets the NPs to tumor vascular cells and into their mitochondria, the other peptide acts as a pro-apoptotic drug. By co-injecting these NPs with iRGD, most of the tumors were eradicated or their development delayed in two glioblastoma mouse models.

Despite the extraordinary rapid development in strategies for nanoparticle conjugation with peptides, relatively little is known about NP behavior in the immune system, which is responsible for maintaining body integrity and preventing external invasion. Bastus et al. described the interaction between murine bone marrow macrophages and gold nanoparticle peptide conjugates. In the presence of conjugates, macrophage proliferation was stopped and pro-inflammatory cytokines were induced. Furthermore, macrophage activation by AuNPs conjugated to different peptides appeared to be rather independent of peptide length and polarity, but dependent on peptide pattern at the nanoparticle surface (Bastus et al., 2009).

#### Proteins/antibodies

In Bionanotechnology, specific functions of proteins such as antibody–antigen detection may also be very useful. Antibodies (Abs) or immunoglobulins are a group of proteins that have a very similar structure with four chains assembled in a Y shaped form, containing two identical domains for antigen recognition (Fab fragment), and two identical domains with effector functions (Fc fragment). The main advantage of Abs or their fragments is that the antigen-binding region is highly specific and different among Abs (Arruebo et al., 2009). Therefore, different specificity can be obtained using distinct Abs. However, attachment of Abs to the surface of NPs can impair this function if the antigen binding sites are sterically blocked upon conjugation. For this reason, the Abs orientation is extremely important to produce effective and bioactive antibody-nanoparticles.

In fact, de la Fuente et al. recently demonstrated that the combination of a good Ab orientation along with the property of a gold nanoprism to generate heat when illuminated with the correct wavelength enable visual detection of carcinoembryonic antigen (CEA) by plasmonic-driven thermal sensing with sensitivities up to the attomolar range in serum samples (Polo et al., 2013).

Immunoassays use the specificity and sensitivity of the antibody-antigen interaction in order to detect and quantify the amount of a specific analyte present in a sample. Effective conjugated antibodies can be used to constitute the desired functionality of the nanocarrier itself for immunoassays (Han et al., 2003). Actually, Putman et al. described the use of immunogold labels as cell-surface markers of human lymphocytes in atomic force microscopy. The AFM images reveal the colloidal gold particles on the cell surface, with and without silver enhancement. Individual immunogold particles are clearly resolved from the cell surface thus determining the location of antigens (Putman et al., 1993).

Ni et al. described an immunoassay readout method based on surface enhanced Raman scattering (SERS). The method exploits the SERS-derived signal from reporter molecules that are co-immobilized with biospecific species on gold colloids. This concept is demonstrated in a dual analyte sandwich assay, in which two different antibodies covalently bound to a solid substrate specifically capture two different antigens from an aqueous sample. The captured antigens in turn bind selectively to their corresponding detection antibodies. The detection antibodies are conjugated with gold colloids that are labeled with different Raman reporter molecules, which serve as extrinsic labels for each type of antibody (Ni et al., 1999).

More recently, Conde et al. developed a highly sensitive probe for *in vivo* tumor recognition with the capacity to target specific cancer biomarkers such as EGFR on human cancer cells and xenograft tumor models. The authors used ~90 nm AuNPs capped by a Raman reporter, encapsulated and entrapped by larger polymers and a Food and drug Administration (FDA) antibody–drug conjugate—Cetuximab (Erbitux®). These smart SERS gold nanoantennas present a high Raman signal both in cancer cells and in mice bearing xenograft tumors and the Raman detection signal is accomplished simultaneously by extensive tumor growth inhibition in mice. This approach seems to be an innovative and efficient theranostics system for both tumor detection and tumor cell inhibition at the same time (Conde et al., 2014a).

QD-Antibody conjugates have also been widely used for preparing bioconjugated QDs for *in vitro* bioassay applications (Goldman et al., 2002; Hua et al., 2006; Tan et al., 2007; East et al., 2011). In fact, Goldman et al. described the preparation and characterization of bioinorganic conjugates made with highly luminescent semiconductor CdSe-ZnS core-shell QDs and antibodies for use in fluoroimmunoassays. QD-antibody conjugates were successfully used in fluoroimmunoassays for detection of a protein toxin (staphylococcal enterotoxin B) and a small molecule (2,4,6-trinitrotoluene) (Goldman et al., 2002).

Concerning magnetic NPs, bioseparation is one of the main applications of MNP-Ab conjugates. In fact, magnetic separation of red blood cells using magnetic microspheres was reported as early as 1977 (Molday et al., 1977). MNPs are used to purify and concentrate different types of analytes in complex samples, such as hormones in biological samples, antibiotics or bacteria in food (Kuo et al., 2012; Svobodova et al., 2012; Xu et al., 2012). One of the best known systems that employ MNPs for separation and concentration is the bio-bar code technology originally described by Nam et al. (2003). In this case, Abs specific for a target protein are functionalized on the surface of MNPs, by sandwiching the target between these MNPs and an amplifier AuNPs that is loaded with a secondary Ab and oligonucleotides. When the specific target is sandwiched between the MNP and the AuNP, magnetic separation of the complexed probes allows for the concentration of the target within the sample. Afterwards, the oligonucleotides are released and detected, giving rise to a substantial amplification of the signal, and therefore lowering the detection limit to attomolar concentrations (Goluch et al., 2006). In fact, the first point-of-care nano-enabled medical diagnostic tool approved by the FDA, known as Verigene System and commercialized by Nanosphere Inc., is based on this biosensing strategy.

MNPs conjugated with Abs are also useful for the development of a new class of diagnostics nanosensors, called magnetic relaxation switches (MRS). MRS have the potential to provide sensitive and selective detection of a variety of molecular interactions with minimal or no sample preparation (Perez et al., 2002). These assays exploit the fact that when MNPs recognize and bind biological targets, they cluster, changing the spin-spin relaxation times of water protons (T2). These changes in T2 between dispersed and aggregated states of the MNPs can be monitored by nuclear magnetic resonance (NMR) (Min et al., 2012). One of the greatest advantages of these biosensors is that they employ radiofrequency radiation which penetrates biological samples regardless of their optical properties, and therefore can be used in complex samples such as blood. Using this technology, Lee et al. reported the first micro NMR biosensor, where tumor cells could be detected employing MNPs functionalized with Abs on microliter sample volumes and in multiplexed format (Lee et al., 2008). Since then, the sensitivity of these biosensors has been greatly improved using other highly magnetic MNPs (Lee et al., 2009), so that molecular profiling of cancer cells obtained by fine-needle aspirates biopsies within 60 min is possible nowadays. Using different markers, the authors reported 96% accuracy for establishing a cancer diagnosis (Haun et al., 2011).

**Figure 6:**
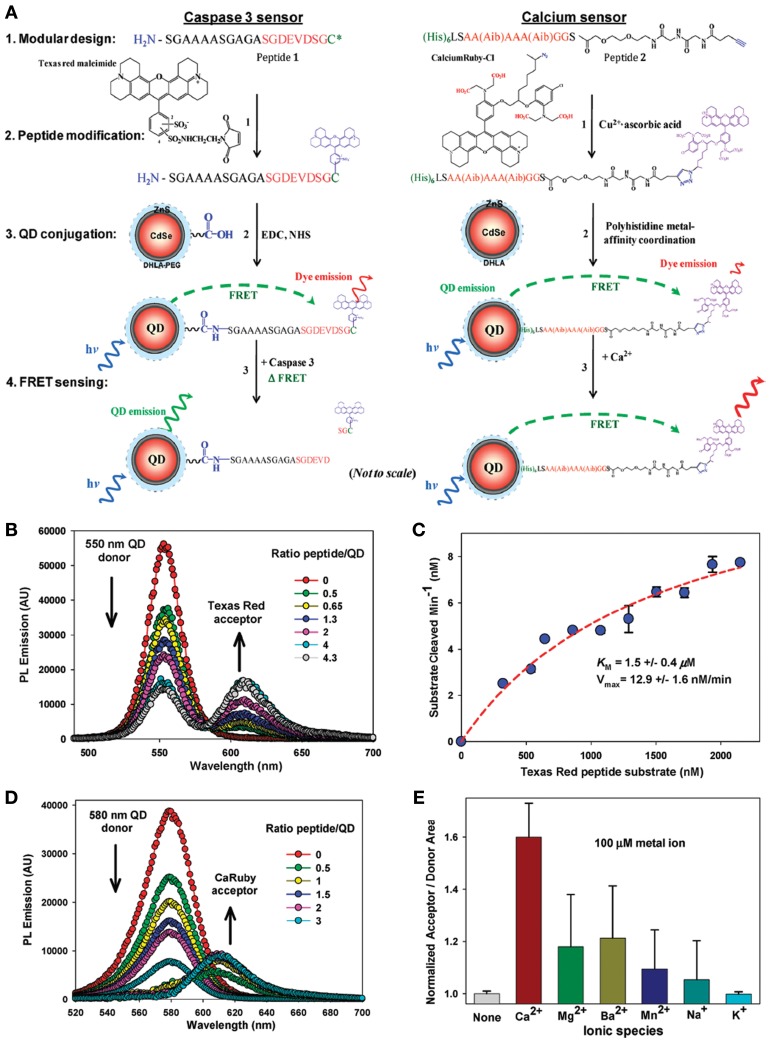
**Quantum dot protein biosensors. (A)** Schematic showing the common design, chemical and sensing elements, including FRET-based biosensors: (1) peptide modularity, (2) peptide labeling, (3) attachment to QDs, and (4) FRET-based sensing for both the caspase 3 proteolytic sensor (left) and Ca^2+^ sensor (right). The 4-pendant carboxyl groups that interact with Ca^2+^ ions are shown in red on the CaRbCl structure. Within the Ca^2+^ sensor peptide sequence, Aib is the synthetic amino acid α-aminoisobutyric acid. Reactive dye structures are shown where appropriate along with the chemical linkages attaching them to the peptides. **(B)** Representative, superimposed spectra collected from 550 nm emitting QD donors surface functionalized with 85:15 DHLAPEG600-COOH/DHLA-PEG750-OMe ligands and covalently conjugated to increasing molar ratios of Texas Red-labeled substrate peptide. Samples excited at 350 nm. **(C)** Proteolytic assay data from exposing a constant concentration of 550 nm emitting QDs conjugated to 4 Texas Red substrate peptides to a constant concentration of caspase 3 enzyme. Derived Km and Vmax values are given. An R^2^ = 0.98 was obtained for the fitting of the curve. **(D)** Representative, superimposed, and deconvoluted spectra collected from 580 nm emitting QD donors self-assembled with increasing CaRbCl-acceptor labeled peptides. Samples were excited at 350 nm. **(E)** Normalized acceptor/donor PL area ratios for 580 nm QDs self-assembled with ~2 CaRbCl-acceptor labeled peptides exposed to selected ionic materials. The ratio from the native unexposed sensor was set to an initial value of 1 for comparison purposes (Prasuhn et al., 2010). Reproduced with permission from Prasuhn et al. (2010), Copyright 2013.

Other proteins have also been successfully conjugated with NPs (Mattoussi et al., 2000; So et al., 2006a,b; Xia et al., 2008; Roullier et al., 2009). Mattoussi et al. first described the electrostatic interactions between negatively charged lipoic acid capped QDs and a positively charged recombinant protein (Mattoussi et al., 2000). Prasuhn et al. also developed a QD protein FRET-based biosensors used as caspase 3 proteolytic and Ca^2+^ sensors (see Figure 6) (Prasuhn et al., 2010).

Similar to nucleic acids, proteins are known for their specific binding interactions and can act together with a wide range of substrates and synthetic analogs. Consequently, high molecular weight peptide ligands show potential for wide biological applications and for stabilization and biofunctionalization of nanocarriers.

#### Enzymes

Enzymes, as highly specialized protein catalysts, are commonly used in biofunctionalization due to their potential in biotechnology and biomedicine, because of the convenience in handling, ease of separation from the reaction mixture and reuse, as well as low product cost. The immobilization in NPs often reduces diffusion limitations and/or enhances the catalytic activity of the enzymes.

An important focus of the research on AuNPs based biosensors is in enzyme electrodes. One recurrent example is glucose biosensors. Zhang et al. (2005b) described the assembly of a gold electrode modified via Au-S bond with AuNPs, where a cystamine monolayer is chemisorbed, thus exposing an array of amino groups. These are further reacted with aldehyde groups of periodate oxidized glucose oxidase via Scchiff base reaction. In this study, the NPs showed to act as conduction intermediates facilitating electron transfer, with little effect on enzyme activity. It was also shown that the sensitivity was improved as well as the affinity for glucose, hence lowering the detection limits.

In another study, MNPs were modified with N-phosphonomethyl iminodiacetic acid for immobilization of urease. Thus, the surface coating was conferred with carboxyl groups to which urease had been immobilized through carbodiimide reaction (Sahoo et al., 2011). The advantage of using MNPs is the possibility of product isolation by a permanent magnet, thus reducing costs. The authors also reported that the thermal stability of the urease was increased, showing that MNPs may be a promising material for storage and enzyme immobilization.

When using enzyme-based biosensors the main concern is reusability of the enzyme. Khoshnevisan et al. reported the use of MNPs to circumvent this dilemma when using cellulase. The enzyme was incubated with the MNPs and binding confirmed by FT-IR. The authors show that immobilization grants higher stability to the enzyme, thus confirming that the use of MNPs in this type of biosensors can be of great benefit (Khoshnevisan et al., 2011).

Imaging QDs *in vivo* is arduous due to the need of an external source of light, which produces strong background autofluorescence from ubiquitous endogenous chromophores. So et al. proposed the ideal QD, where it would emit light with no requirement for external excitation (So et al., 2006b). By modifying QDs with *Renilla reniformis* luciferase the authors discard the need of external excitation due to the phenomenon of bioluminescence resonance energy transfer (BRET). BRET occurs naturally and it is analogous to FRET, but the donor energy comes from a chemical reaction catalyzed by the donor enzyme. The polymer coated CdSe/ZnS core-shell QDs dotted with carboxylate groups were incubated with *R. reniformis* luciferase, where through carbodiimide reaction the amino groups of the enzyme were coupled to the carboxylates. Thus, with a simple modification the authors were able to mimic the natural BRET system with self-illuminating QDs (So et al., 2006b).

#### Carbohydrates

Carbohydrates are, together with nucleic acids and proteins, important molecules for life. Much is already known about the structure, interactions and function of nucleic acids and proteins, however, the role of carbohydrates in the cell is less clear (de la Fuente and Penades, 2006). A characteristic feature of the biological interactions where carbohydrates are involved is their extreme low affinity that has to be compensated by multivalent presentation of the ligands. Although individual carbohydrate interactions are relatively weak, nature utilized multivalent interactions between the cell surface ligands and their biological receptors to modulate biological events such as the ones related to cell adhesion, normal tissue growth and repair, viral/bacterial infection, signaling transduction, trapping of leucocytes, and cancer transfer. So the decoding of carbohydrate interactions opens up the possibility to employ nanoparticles in diagnostics and/or therapy (Dong, 2011). In fact, the unique physical, chemical and optical properties of the nanocarriers with carbohydrate coating comprise a series of advantages that range from ensuring water solubility, biocompatibility and stability to targeting properties (Garcia et al., 2010).

**Figure 7:**
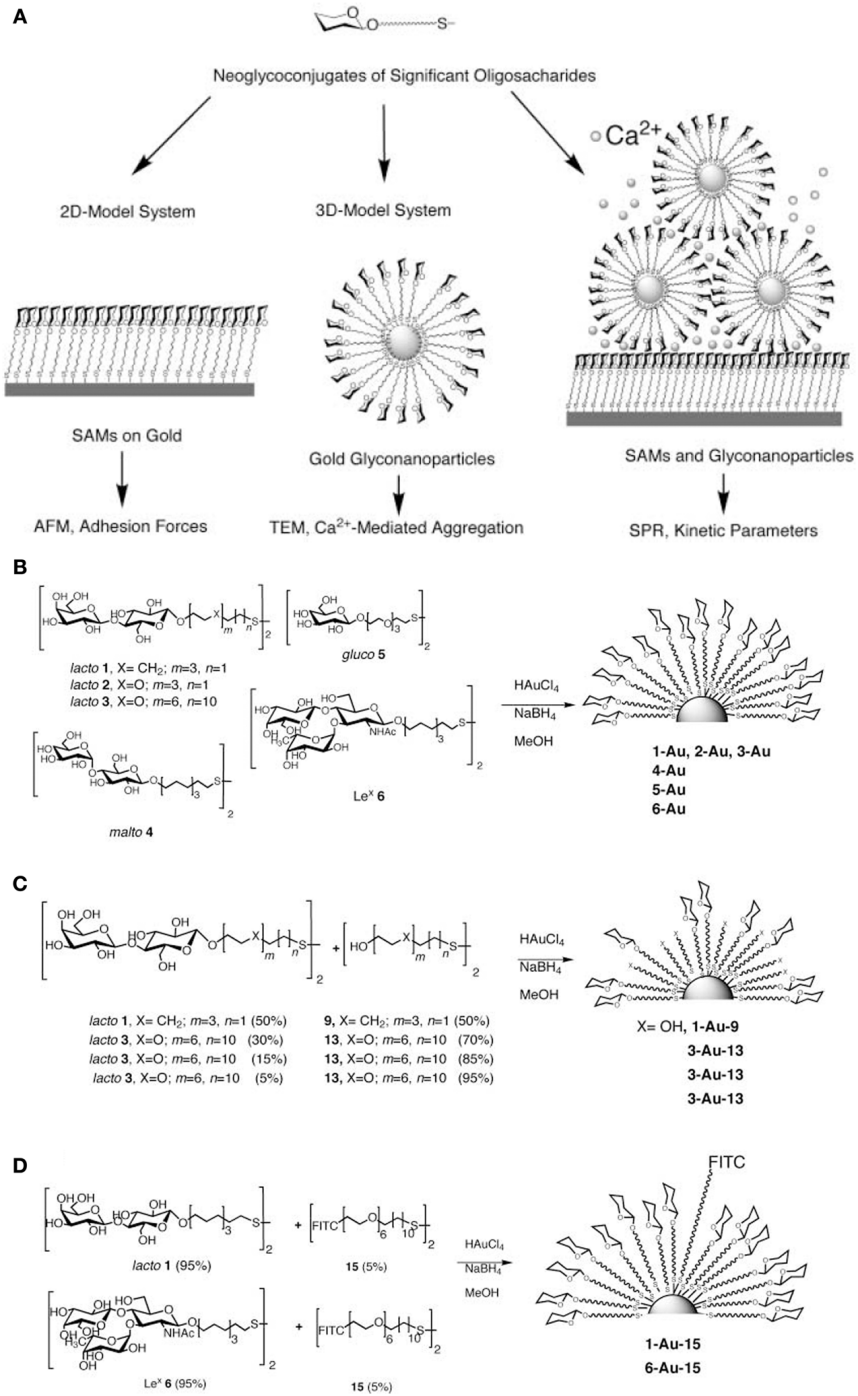
**Gold glyconanoparticles. (A)** Strategy for studying carbohydrate ± carbohydrate interactions based on 2D and 3D models that mimic carbohydrate presentation at the cell surface. Preparation of **(B)** glyconanoparticles; **(C)** hybrid glyconanoparticles; **(D)** fluorescence glyconanoparticles (Barrientos et al., 2003). Reproduced with permission from Barrientos et al. (2003), Copyright 2013.

Among them, gold glyconanoparticles (glycoNPs) have drawn attention owing to their well-defined features, such as water-soluble carbohydrate-functionalized nanoclusters with a promising potential for chemical glycobiology, biomedicine, diagnostics and clinical applications. In the last 10 years, Penades and co-workers have extensively reported a pioneer integration of a glyconanotechnology strategy based on the use of nanoparticles to study and evaluate carbohydrate–carbohydrate, carbohydrate–protein interactions (Figure 7) (de la Fuente et al., 2001, 2006; Barrientos et al., 2003; de la Fuente and Penades, 2004, 2006), which could be used as potential tools in anti-adhesive therapy (Rojo et al., 2004), for cell–cell adhesion studies (de la Fuente et al., 2005), prevention of pathogen invasion (Reynolds et al., 2012) and for exploring blood–brain barrier permeability via neuropeptide conjugation (Frigell et al., 2014).

Smaller carbohydrates, such as lactose, glucose and mannose (Otsuka et al., 2001; Reynolds et al., 2006; Schofield et al., 2007; Martinez-Avila et al., 2009) can be thiolated for attachment to AuNPs via ligand exchange. These nanoparticles may be useful as sensitive colorimetric probes for a variety of metal ions. Mannose and lactose have also been used for the reduction of gold salts and stabilization of the nanoparticles. Schofield et al. have shown that thiolated carbohydrate derivatives can be readily assembled on silver and gold NPs. These metal glycoNPs can be used to develop aggregation based colorimetric bioassays (Schofield et al., 2006).

Magnetic glycoNPs with unique properties have also been reported, although in a more limited number (El-Boubbou and Huang, 2011; Marradi et al., 2013). Once carbohydrates are attached on the MNPs, it is crucial that they retain their biological activity. To explore this, plant lectins can be used, as their interaction with carbohydrates is highly selective. The clustering of the MNPs due to the selective recognition of the lectin can be detected using MRS assays (Moros et al., 2010).

Carbohydrates can also be used to target different cells and/or enhance the cellular uptake of NPs in a highly specific way. For instance, Moros et al. functionalized MNPs with glucose and galactose using EDC and studied their interaction with Vero cells *in vitro* (Moros et al., 2012). Although these monosaccharides share the same chemical formula, except for the spatial conformation of the hydroxyl group in C-4, the cell entrance pattern was completely different. While MNPs-glucose entered all throughout the cell, MNPs-galactose remained predominantly in the cell periphery. By preparing a library of MNPs functionalized with different monosaccharides, El-Boubbou et al. were also able to detect, differentiate cancer cells and quantitatively profile their carbohydrate binding abilities by MRI (El-Boubbou et al., 2010).

Carbohydrates have been also conjugated to QDs (Chen et al., 2003; Osaki et al., 2004; Kikkeri et al., 2009; Cai et al., 2012; Yang et al., 2012a). For example, Kikkeri et al. synthesized PEGylated QDs capped with D-mannose, D-galactose, and D-galactosamine to study specific carbohydrate-protein interactions *in vitro* and *in vivo*. These QD-carbohydrates were produced through covalent coupling by 4-maleimidopropanoic acid NHS ester and used for *in vitro* imaging and *in vivo* liver targeting (Kikkeri et al., 2009). Shinchi et al. also developed glycol-QDs by preparing stable sugar-chain-immobilized fluorescent nanoparticles (CdTe/CdS core/shell QDs functionalized with sugar-chain-ligand conjugates, β-galactose- and α-glucose) and their application to the analysis of sugar-chain-protein interactions and cellular imaging (Shinchi et al., 2012).

### Biomolecule coupling strategies

Functionalization of NPs with biomolecules has to face several hurdles and surface modifications can have significant impacts on their physical-chemical properties and therapeutic efficacy, once they might alter surface charge, size, hydrophobicity, and targeting skills. One of the biggest challenges is that NPs need to remain stable in solution while the conjugation takes place. However, many NPs may precipitate while being activated, as their stability depends on a delicate balance between attractive and repulsive forces, which can be modified when using different chemicals for their biofunctionalization. Moreover, due to the huge amount of different NPs and biomolecules reported so far, there are no standardized protocols for NP functionalization. Therefore, the choice of a coupling strategy depends on the stability of the NP, the functional groups, the bioconjugation conditions (pH, temperature, ionic strength, solvent choice, structure of the surfactant) and the biomolecule to attach, among others. Finally, depending on the conjugated biomolecule, it is important to control the orientation, so that the biomolecule remains active once conjugated to the NP.

Biofunctionalization can be achieved using several techniques, such as physical adsorption and electrostatic binding, specific affinity recognition, and covalent coupling, each of which has its own advantages and disadvantages. In this section of the review, the several coupling strategies for biofunctionalization of gold, MNPs and QDs will be examined.

#### Covalent strategies

Covalent coupling provides stable and strong binding of the biomolecules to the NPs. Most proteins have amine groups in their surface, so they can be directly conjugated to NPs containing reactive groups such as aldehydes, epoxides or anhydrides (Fuentes et al., 2005; Lu et al., 2008).

On the other hand, coupling of NPs that exhibit amine groups with molecules containing aldehydes or epoxides can also be used. However, some biomolecules such as antibodies, oligonucleotides, carbohydrates or peptides do not include these functional groups, and should be modified prior to the conjugation (Nobs et al., 2004). For instance, carbohydrates present in some antibodies can be oxidized using periodate to generate aldehyde groups that can react with the amino groups present on the NPs surface (Fuentes et al., 2005). Nevertheless, chemical modification may compromise biomolecules' activity, so one of the most frequent ways to conjugate molecules to the NPs is using linker molecules.

***EDC coupling reaction.*** 1-Ethyl-3-(3-dimethylaminopropyl)-carbodiimide (EDC) is a zero-length crosslinking agent used to couple carboxyl or phosphate groups to primary amines, which may react with a carboxyl group of a biomolecule, forming an amine-reactive O-acylisourea intermediate. Addition of sulfo-NHS stabilizes the amine-reactive intermediate by converting it to an amine-reactive sulfo-NHS ester. The O-acylisourea intermediate may also react with an amine on a second biomolecule, producing a conjugate of the two biomolecules joined by a stable amide bond. This crosslinker has been used in diverse applications, such as conjugation of carboxyl to amine groups in peptides and proteins, forming amide bonds in peptide synthesis, attaching haptens to carrier proteins and form immunogens, labeling nucleic acids through 5′ phosphate groups and creating amine-reactive NHS-esters of biomolecules (Grabarek and Gergely, 1990).

One of the main advantages of EDC coupling is water solubility, which allows direct bioconjugation without prior organic solvent dissolution. On top of that, the excess of reagents and by-products can be easily removed by dialysis or gel-filtration (Sheehan et al., 1965). However, the coupling reaction has to be carried out fast, as the reactive ester that is formed can be rapidly hydrolyzed in aqueous solutions. To increase the stability of this active ester, N-hydroxysuccinimide (NHS) or N-hydroxysulfoxuccinimide (sulfo-NHS) can be used (Jang and Keng, 2008). Key parameters that should be controlled when using EDC are pH (as hydrolysis is largely dependent on pH), the amount of EDC so that NPs do not aggregate due to loss of electrostatic repulsive forces between NPs, and the ratio EDC/NHS (Nakajima and Ikada, 1995; Sam et al., 2009; Shen et al., 2009).

**Figure 8:**
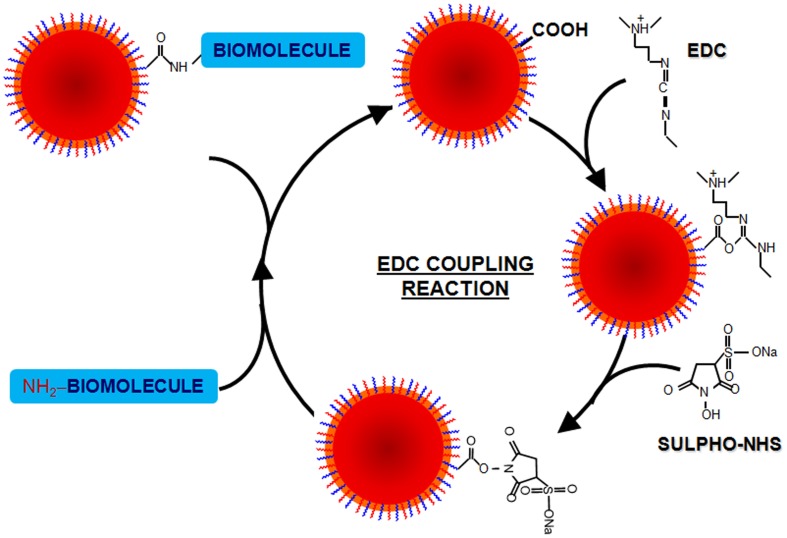
**EDC coupling reaction**. The 1-ethyl-3-(3-dimethylaminopropyl)-carbodiimide (EDC) is a zero-length crosslinking agent used to couple carboxyl groups to primary amines. In the presence of N-hydroxysulfosuccinimide (Sulfo-NHS), EDC can be used to convert carboxyl groups to amine-reactive Sulfo-NHS esters. The addition of Sulfo-NHS stabilizes the amine-reactive intermediate by converting it to an amine-reactive Sulfo-NHS ester, thus increasing the efficiency of EDC-mediated coupling reactions. Excess reagent and crosslinking by products are easily removed by washing with water. Once EDC is water soluble, the crosslinking can be done under physiologic conditions without adding organic solvent.

Using this protocol almost all kinds of molecules (i.e., enzymes, antibodies, peptides, DNA, fluorophores, etc.) may be attached to the nanoparticle surface without prior modification (see Figure 8) (Pandey et al., 2007; Susumu et al., 2007; Rostro-Kohanloo et al., 2009; Conde et al., 2012a; Lavilla et al., 2012). For instance, using the EDC chemistry, Weissleder and coworkers created a library of MNPs decorated with different synthetic small molecules for the development of magnetofluorescent reporters (Weissleder et al., 2005). Using these fluorescent MNPs it was possible to screen against different cell types or among different physiological states of a cell line. On the other hand, Sanz et al. have reported the effect of biofunctional spacers, such as thiolated PEG chains on the loading of RNA molecules and a positive peptide functionalized by EDC coupling reactions on the surface of AuNPs (Sanz et al., 2012). Lin and coworkers also used EDC to attach CH_3_O-PEG-NH_2_ to different types of carboxylated NPs (MNPs, QDs) demonstrating that adjusting the ratio EDC/NP it was possible to prepare NPs with 0, 1, or 2 attached PEG molecules (Lin et al., 2008). Similarly, Parak and co-workers also used EDC to attach NH_2_–PEG–NH_2_ molecules, varying the molecular weight of the polymer on the surface of AuNPs (Sperling et al., 2006; Pellegrino et al., 2007). The covalent attaching of biofunctional short PEG molecules to the polymer shell produces very stable particles in electrolytic solution. This approach results in stable water-soluble AuNPs (Sanz et al., 2012) and QDs (Ballou et al., 2004) with functional groups, e.g. −COOH or −NH_2_ on the free ends of PEG molecules. By controlling the EDC ratio, aggregation was prevented. Dhar et al. have exploited the rapid intracellular uptake of AuNPs to deliver and activate cisplatin and achieve efficient cytosolic delivery of platinum(IV) prodrug to lung cancer cells. The AuNPs used in this study were functionalized with thiolated oligonucleotides containing a terminal dodecyl amine for conjugation with a platinum(IV) compound capable of being tethered to an amine functionalized DNA-AuNP surface via amide linkages (Dhar et al., 2009).

Similarly, conjugation of biomolecules to QDs through coupling reactions with reactive functional groups presented on QDs surface is another common strategy to prepare QD bioconjugates. Actually, carboxylic acid groups can also be added to QDs' surface and subsequently conjugated to biomolecules with primary amine groups through EDC coupling reactions (Cai et al., 2006; Hua et al., 2006; Choi et al., 2009; Wu et al., 2009a; East et al., 2011).

One disadvantage of this type of chemistry is that the presence of both carboxylates and amines on one of the biomolecules to be conjugated with EDC can result in self-polymerization and consequently, loss of effectiveness. For instance, peptides usually contain both types of groups, so if EDC is added in the presence of them, peptides can polymerize. However, Bartczak et al. have used this strategy to coupling of peptides to AuNPs in a one-pot way. The authors have shown that the concentration, reaction time, and chemical environment are all critical to achieving the formation of robust, peptide-coated colloidal nanoparticles without aggregation (Bartczak and Kanaras, 2011). Another way to avoid polymerization of the biomolecule is to eliminate the excess of EDC before adding the biomolecule to the NPs solution by magnetic separation in the case of MNPs or gel-filtration for other NPs.

**Figure 9:**
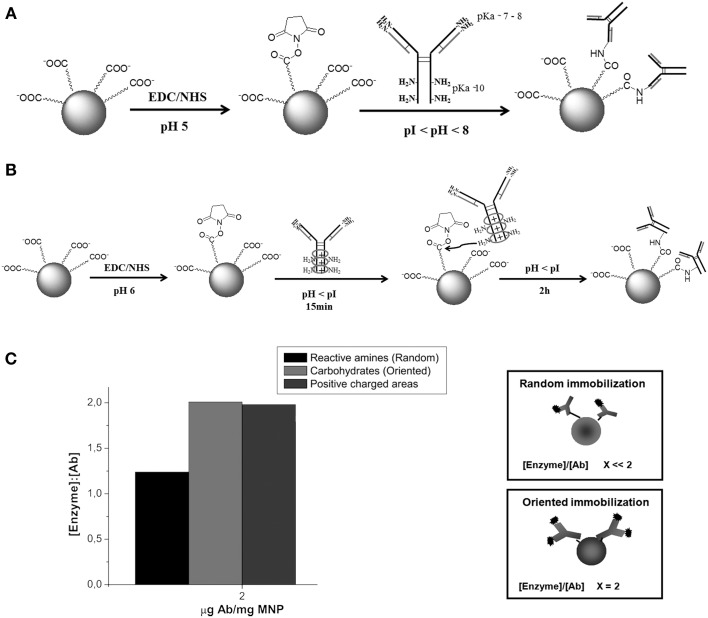
**Highly active magnetic nanoparticle-antibody conjugates. (A)** Two-step immobilization mechanism proposed when using Ab that bind to the MNPs through the most reactive amines—random immobilization. **(B)** Covalent attachment via the polysaccharide moieties of the antibody to the MNPs—oriented immobilization. **(C)** Capacity to capture HRP of the anti-HRP anchored to MNPs by different orientations. The protein content of all anti-HRP-functionalized MNPs was similar (2 μg Ab per mg MNP) (Puertas et al., 2011). Reproduced with permission from Puertas et al. (2011), Copyright 2013.

Despite the simplicity of this technique, that does not require prior chemical modification of the biomolecule, it does not guarantee an oriented immobilization in the case of biomolecules with greater structural complexity, such as antibodies. Due to the poor stability of the reactive ester, neutral pH is traditionally used to link covalently antibodies to carboxylated-NPs. At this pH, immobilization mainly occurs through direct covalent binding of the most reactive amine groups of the antibody. Unfortunately, these are the terminal amine moieties of the four Ab polypeptide chains (pKa around 7–8), which are all located in the antigen-binding domain (Puertas et al., 2011). Recently, Puertas et al. described a smart approach that takes advantage of the existing kinetic differences among ionic adsorption processes and covalent reactions in order to assure the oriented covalent attachment of the Ab using EDC chemistry. Briefly, it requires the selection of the best incubations conditions (pH, ionic strength) to promote a fast ionic adsorption of the Ab due to the negative charges of the carboxylic groups of the NP. This ionic adsorption makes possible the orientation of the Ab on the NP surface before irreversible covalent bond formation (Figure 9) (Puertas et al., 2011). Initially, the authors optimized this two-step strategy for magnetic NPs but they have recently extended application for functionalization of other nanostructured materials, such as gold nanoprisms and carbon nanotubes (Polo et al., 2013).

**Figure 10:**
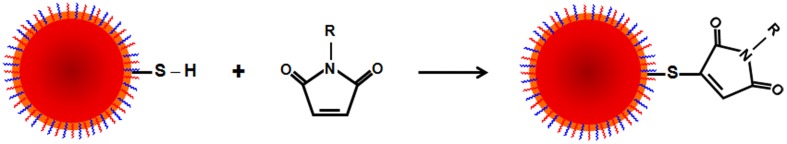
**Maleimide coupling reaction**. Maleimide reacts with free sulfhydryl group(s), forming stable thioether linkages, at physiological pH. It is useful for bioconjugation of proteins with −SH groups and the coupling of two thiols to form a disulfide linkage.

***Maleimide coupling.*** Maleimide can be used to conjugate primary amines to thiols (Brinkley, 1992) (see Figure 10). The use of maleimide for modification of sulfhydryl groups has been extensively described in the literature (Means and Feeney, 1990). Reaction with sulfhydryl groups generates a stable 3-thiosuccinimidyl ether linkage and occurs normally at pH 6.5–7.5. One of the main limitations is that the maleimide ring may hydrolyze in aqueous buffer to a non-reactive cis-maleamic acid derivative over long reaction times or at pH > 8.0. Nevertheless, this type of conjugation shows a lot of potential for a great number of biomolecules that bear reactive thiol or amino groups. This may eventually lead to non-specific bonds and crosslinking between functionalized nanoparticles since a single biomolecule may have several thiol groups (Means and Feeney, 1990; Brinkley, 1992).

Maleimide coupling has been used to conjugate several biomolecules to AuNPs, such as peptides (Oh et al., 2010a; Ravi et al., 2012), chemotherapeutic agents (Hwu et al., 2009), dyes (Zhu et al., 2012a), and DNA (Lee, 2011). In fact, Ba et al. presented a versatile and controlled route to immobilize AuNPs on the surface of living cells, while preserving the sensing and optothermal capabilities of the original colloid, by chemically anchoring the nanoparticles to phospholipids in liposomes via maleimide-thiol reactivity (Ba et al., 2010).

Maleimide coupling was also used to couple DNA (Dubertret et al., 2002), PNAs (Srinivasan et al., 2006), proteins (Wolcott et al., 2006; Bonasio et al., 2007; Zhou et al., 2007), and antibodies into QDs (Diagaradjane et al., 2008). To address biocompatibility issues of QDs, Dubertret et al. encapsulated individual nanocrystals in phospholipid block-copolymer micelles conjugated to DNA and demonstrated their function as fluorescent probes (Dubertret et al., 2002). Bonasio et al. also reported the specific and covalent labeling of QDs with a membrane protein and organic fluorophores (Bonasio et al., 2007).

Similarly, MNPs can also be functionalized using the maleimide coupling reaction with PEG (Kuhn et al., 2006), DNA (Nam et al., 2004) or even drugs, such as chlorotoxin (Kievit et al., 2010). Concerning antibodies, this chemistry could be also used with thiol or amino groups on the nanoparticle surface (Lee et al., 2007; Haun et al., 2010). Regarding a thiolated NP, antibodies would bind through their most reactive amine groups and, as previously explained, this could lead to a random orientation with partial loss of the Ab's biological activity. Instead, maleimide chemistry used with aminated NPs ensures an oriented binding through thiol groups of the Ab. However, as in Abs sulfhydryls are oxidized as disulfides. So it is necessary to selectively reduce the disulfides at the hinge region by a reducing agent (i.e., 2-mercapthoethylamine, mercaptoethanol, dithiotreitol, thiopropyl-agarose). This chemical modification can also be combined with fragmentation of the IgG by the use of proteolytic enzymes (i.e., pepsine, ficin) in order to conjugate small Ab fragments such as F(ab′)2 and Fab′.

**Figure 11:**
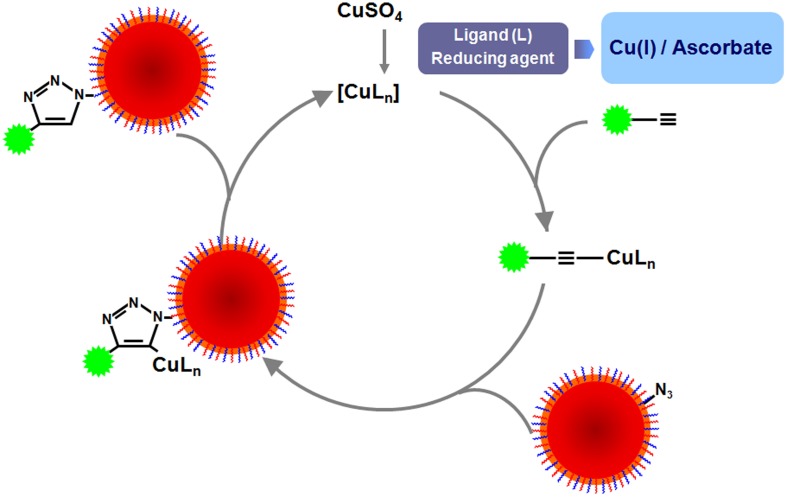
**Click chemistry reaction**. The copper-catalyzed cycloaddition of azides and alkynes (CuAAC) developed for click chemistry joins an organic azide (N_3_) and alkyne together producing a mixture of 1,4- and 1,5-triazoles.

***Click-chemistry reaction.*** The copper(I)-catalyzed azide-alkyne cycloaddition (CuAAC) click reaction has been recognized as a facile and versatile chemistry for bioconjugation. Azides and alkynes are highly energetic functional groups with particularly narrow distributions of reactivity. Thanks to their weak acid-base properties, they are nearly inert toward biological molecules and toward the reaction conditions found inside living cells. The azide groups are easy to introduce into organic compounds by both nucleophilic and electrophilic processes. One of the most common bioconjugation of azides is the copper catalyzed azide-alkyne cycloaddition (CuAAC) (Wang et al., 2003) (see Figure 11). This reaction features an enormous rate acceleration of 10^7^–10^8^ compared to the uncatalyzed 1,3-dipolar cycloaddition (Himo et al., 2005). This reaction has also been termed the “cream of the crop” of click reactions and is surely responsible for the tremendous popularity of the “click” concept and many simply associate “click chemistry” to mean triazole formation between an azide and alkyne. The reaction occurs at room temperature, showing a high degree of solvent and pH insensitivity, and high chemoselectivity (the azide and alkyne are inert to react with numerous functional groups under the typically mild reaction conditions). In fact, the reaction succeeds over a broad temperature range, is insensitive to aqueous conditions and occurs in a pH range between 4 and 12 (Hein and Fokin, 2010; Le et al., 2010). The copper catalyzed azide-alkyne cycloaddition occurs between an organic azide and a terminal acetylene. The cyclic product is a triazole. The copper catalyst allows the reaction to proceed at room temperature and confers regioselectivity (a reaction in which one direction of bond making or breaking occurs preferentially over all other possible directions), with the 1,4 regioisomer being the only product. The reaction start by the incubation with a mixture of copper(II) (e.g. copper(II) sulfate) and a reducing agent (e.g. sodium ascorbate) to produce Cu(I) *in situ* (Meldal and Tornoe, 2008; Hong et al., 2009).

Click chemistry sometimes refers to a group of reactions that are fast, simple to use, easy to purify, versatile, regiospecific, and give high product yields. However, the click reaction has a number of limitations. First, like with any cycloaddition, if the azide group is too electron deficient, then it will not undergo the reaction. In other words, the ground state configuration of the azide is far too low to interact with the terminal alkyne (Hein et al., 2008). Secondly, a more common problem is alkyne homocoupling. This phenomenon occurs when an alkyne reacts with a second alkyne instead of the azide. This process can be minimized by using a sterically bulky base that stabilizes the reactive intermediates of the homocoupling reactions. The Cu(I) saturation is rare but can also be a problem, once the alkynes may chelate the Cu(I)-acetylide complex intermediate that contact with the azide group (Hein et al., 2008). Besides these limitations, one of the most obvious disadvantages is the requirement of a copper catalyst. In fact, an excessive intake of copper can lead to drastic consequences for the human body (e.g. hepatitis, neurological disorders, kidney diseases and Alzheimer's disease) (Wang and Guo, 2006; Hein et al., 2008).

Generally, the click-chemistry reaction has been used to couple AuNPs to proteins (Zhu et al., 2012b), enzymes (Brennan et al., 2006; Kim et al., 2010), fluorophores (Voliani et al., 2011), polymers (Boisselier et al., 2008; Zhang et al., 2009), and other small molecules (Fleming et al., 2006). For example, following click-chemistry reaction Fleming et al. were able to conjugate to AuNPs several different alkyne derivatives, such as ferrocene, aniline and PEG (Fleming et al., 2006).

Alkyne-functionalized AuNPs have been also extensively used to detect metal ions in aqueous solutions, such as Cu^2+^, using click chemistry. This method allows visualization by naked eye of the presence of Cu^2+^ ions by the aggregation of AuNPs as a result of the Cu(I)-catalyzed conjugation between the two functional groups (Zhou et al., 2008b; Xu et al., 2010; Lin et al., 2012).

Another common type of nanoparticles used for click-chemistry bioconjugation is QDs. QDs need to be coated to other chemical species if they are to be used as biomarkers, therapeutic agents or sensors. In fact, water soluble and water QDs have been successfully coated with polymers via click-chemistry reactions (Beaune et al., 2011; Janczewski et al., 2011; Lai and Guan, 2011; Petryayeva and Krull, 2012; Zhang et al., 2012a). Jańczewski et al. reported the use of click-QDs by producing water solubilization of hydrophobic CdSe/ZnS QDs using amphiphilic polymeric coatings. The authors described the preparation of acetylene- and azide-functionalized QDs for “click” chemistry. The method is universal and applicable to any type of nanoparticle stabilized with hydrophobic ligands able to interact (in water) with the alkyl chains present in the coating (Janczewski et al., 2011).

Interestingly, Hao et al. reported a method for labeling viruses via copper-free click chemistry to QDs. The authors linked virions modified with azide to QDs capable of realizing single-virion tracking, laying the foundation for long-term dynamic visualization of virus infection process (Hao et al., 2012).

Although click chemistry does not appear to be a major type of chemistry for MNPs, some interesting examples can be found in the literature. For instance, Santra and coworkers reported the creation of novel polymeric-metallic nanocomposites when assembling alkylated IONPs with azide polymer fluorescent NPs, obtaining a fluorescence material with enhanced magnetic properties for MRI (Santra et al., 2009). The first example of click MNPs for *in vivo* applications was reported by Bhatia and coworkers (von Maltzahn et al., 2008). Fluorescent MNPs functionalized with a tumor-targeting peptide (Lyp-1) *via* click chemistry were able to stably navigate the systemic circulation, extravasate into tumors and penetrate into the interstitial space to specifically bind to receptors on tumor cells. Weisledder group also reported the introduction of ^18^F onto azide-modified MNPs using click-chemistry for *in vivo* PET imaging (Devaraj et al., 2009).

#### Non-covalent strategies: physical interactions

Physical interactions include electrostatic, hydrophobic and affinity interactions. These interactions have several advantages, such as the ease of functionalization, speed of binding and that neither the biomolecules nor the NPs must be modified in case of electrostatic and hydrophobic interactions. However, conjugation is less stable and reproducible when compared to covalent methods. Moreover, it is difficult to control the amount and orientation of bound molecules.

***Ionic coupling.*** Ionic adsorption provides a simple and straightforward method to functionalize NPs with biomolecules. In fact, biological and polymeric species with an opposite charge can be coupled to NPs (Conde et al., 2012a) or between different opposite charged NPs (Liu et al., 2012). Ionic binding rate mainly depends on the amount of charges present on the NPs and the biomolecules, as the binding is made by multiple point (multipunctual). Therefore, when binding complex molecules such as antibodies or proteins, the isoelectric point should be considered, as their net charge would depend on it.

Ionic coupling has been traditionally used to adsorb proteins to NPs (Brewer et al., 2005; Hong et al., 2006; Reed and Metallo, 2010; Guo et al., 2011; Brancolini et al., 2012; Strozyk et al., 2012), as some proteins such as serum albumin can stabilize NPs by preventing aggregation (Brewer et al., 2005). Moreover, proteins can be adsorbed to NPs to increase cellular uptake or specificity toward tumor cells (Chang et al., 2012). Negatively charged hyaluronic acid (HA) was also used to self-assemble onto the positively charged QDs through ionic interactions. For this, Bhang et al. developed a simple and novel electrostatic coupling method, which provides a HA-QD conjugate with cancer targeting efficiency to use in diagnostic and imaging applications. These conjugates were also effective for fluorescence staining of lymphatic vessels *in vitro* and *in vivo* (Bhang et al., 2009).

Despite the ease of this conjugation method, the native structure of the adsorbed proteins may be affected (Lacerda et al., 2010), which could ultimately result in loss of biological activity or even cellular toxicity (Vertegel et al., 2004; Deng et al., 2011).

**Figure 12:**
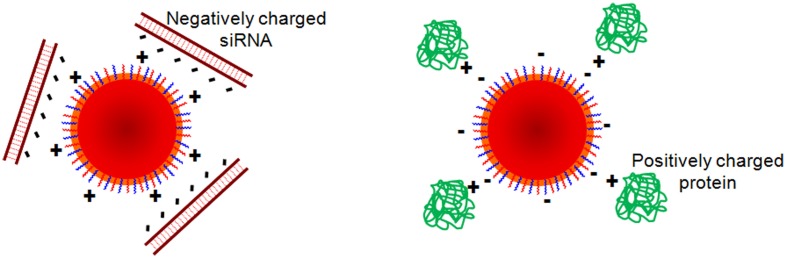
**Ionic coupling**. Coupling of negatively charged siRNA (left) and of positively charged proteins (right) to negatively or positively charged AuNPs.

Other examples of biological applications are the coupling of negatively charged DNA (Thomas and Klibanov, 2003; Ghosh et al., 2008; Conde et al., 2012b) or siRNA (Elbakry et al., 2009; Guo et al., 2010; Conde et al., 2012a; Zhao et al., 2012a) to positively charged NPs (Figure 12) (Huschka et al., 2012). In fact, Conde et al. reported functionalization of siRNA by ionic coupling to a positively charged layer formed by quaternary ammonium groups (R_4_N^+^). Ionic interactions between the negatively charged siRNA backbone (via phosphate groups) and quaternary ammonium positively charged groups ensured binding of siRNA onto the AuNPs' surface for the whole pH range. Using a hierarchical approach including three biological systems of increasing complexity, *in vitro* cultured human cells, *in vivo* freshwater polyp (*Hydra vulgaris*) and *in vivo* healthy mice model, these authors identified the most adequate nanoparticles to efficiently transport siRNAs. The results evidenced the importance of a correct design in the functionalization of nanoparticles for biological applications, in particular for complex animal systems, such as mice. The ionic linkage of siRNA on the AuNPs showed efficiency in cells and in *Hydra*. However, only a covalent bond ensured an active and efficient RNAi mechanism in mice (Conde et al., 2012a). Similarly, Li et al. reported the development of QDs-DNA complexes that are disrupted and DNA released by glutathione (GSH) at intracellular concentrations (Li et al., 2008).

As extensively reviewed by others (Montenegro et al., 2013), conjugation of Abs to NPs may be made with covalent immobilization techniques such as those mentioned before, but also with non-covalent strategies. The most common technique is the electrostatic adsorption of Abs by charge interaction to opposite charged NPs. This ionic adsorption is directly related to the Abs isoelectric point, pH at which they are neutral. Since it depends mainly on the number of charged groups, the Abs immobilized region will be where the greatest number of charges are present (Jung et al., 2008). This method, although of easy implementation, shows several disadvantages. The main concern is the weak pH dependent interaction between the Ab and NP. Any changes to the pH and/or ionic strength may incur in desorption of the Abs molecules. Additionally, the heterogeneous charge distribution and the unexploited charged groups of the Abs can promote non-specific adsorption to matrix proteins, for example competitive displacement caused by serum proteins (van der Voort et al., 2004; Murcia and Naumann, 2005).

**Figure 13:**
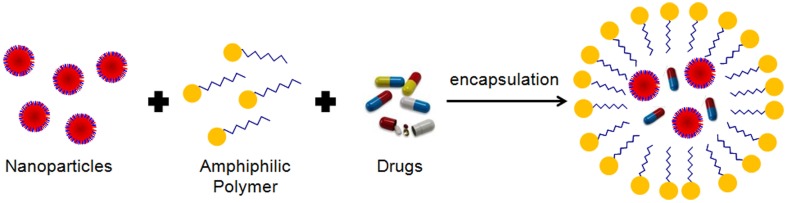
**Hydrophobic coupling**. Use of amphiphilic polymers as nanoparticle and drug delivery moieties.

***Hydrophobic coupling.*** Hydrophobic interactions have been widely exploited to attach lipophilic drugs to NPs, from where the drug might be released once inside the cells (Wahajuddin and Arora, 2012). For instance, docetaxel has been easily adsorbed to the oleic acid layer that surrounded hydrophobic MNPs prior to their encapsulation in a polymeric vesicle, providing a controlled drug release profile for a month (Ling et al., 2011). Encapsulation of drugs can also be done adsorbing them to hydrophobic polymers or cyclodextrins for instance (Yallapu et al., 2011) (see Figure 13).

Similarly, hydrophobic molecules such as fluorophores can be coupled to NPs. Foy and coworkers coated hydrophobic NPs with amphiphilic polymers, to which they adsorbed five different dyes to determine MNP biodistribution using a mouse xenograft breast tumor model (Foy et al., 2010). Moreover, Kim et al. developed AuNPs functionalized with water-soluble zwitterionic ligands from kinetically stable complexes with hydrophobic drugs and dyes, which are efficiently released into cells (Kim et al., 2009). This coupling method minimizes changes to the surface and allows the creation of NP with dual properties, such as optical and therapeutic, in an easy way.

Proteins and Abs can also be adsorbed to NPs *via* hydrophobic interactions. However, they often suffer from denaturation, leading to poor reproducibility. Hydrophobic interactions with the hydrophobic surfaces of proteins or Abs force a change in the native structure because of exposure of its inner region, which could ultimately results in loss of activity (Zuo et al., 2010; Shemetov et al., 2012). Moreover, regarding ionic binding, controlling the orientation or the amount of bound molecules is difficult to achieve.

***Biotin-avidin system.*** For NPs conjugation, factors such as solubility, charge and all the aforementioned functional groups confer the biotin a relevant importance (Aslan et al., 2004). Biotinylation of NPs and biological molecules nowadays is fairly common as biotin can be synthesized to have a distal amine, thiol, carboxyl and other functional groups, simplifying the conjugation (Hermanson, 2008). However, to avoid a random immobilization of structural complex biomolecules such as proteins, it is compulsory to achieve a site specific biotinilation. Concerning antibodies, for example, biotinilation must be carried out within its Fc region via the carbohydrates moieties or via thiols obtained after reduction of the disulfides located in the hinge region (Cho et al., 2007).

**Figure 14:**
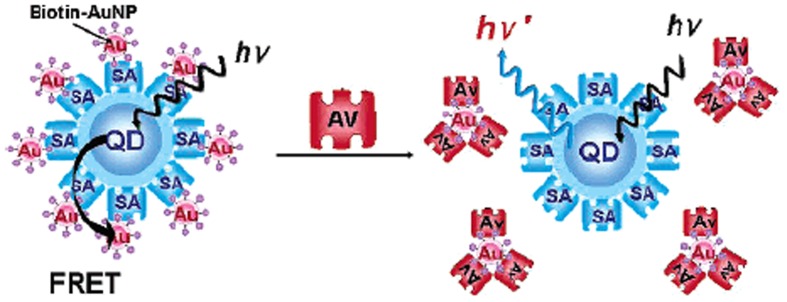
**Biotin-avidin system**. Schematic of inhibition assay method based on the photoluminescence quenching of SA-QDs by Biotin-AuNPs. SA denotes the streptavidin immobilized on the surface of QDs, and Av is the externally added avidin (Feng et al., 2013). Reproduced with permission from Feng et al. (2013), Copyright 2013.

Genetic engineering has also improved biotinylation by recombinantly introducing biotin labeling sites into fusion proteins (Cronan, 1990; Cull and Schatz, 2000). Nowadays several companies offer biomolecules and NPs modified with biotin or avidin species. The high-affinity of the avidin-biotin interaction (*K_d_* around 10^−14^) has made it perfect for the development of NPs-based biosensors. The main method of bioconjugation using avidin-biotin chemistry comprises the functionalization of NPs with avidin, for later incubation with a biotinylated molecule. Since avidin and its variants are zwitterionic molecules, they can be subject to electrostatic adsorption to negatively charged nanoparticles (see section Non-Covalent Strategies: Physical Interactions). The vast number of publications that apply biotin-avidin interaction for bioconjugation shows the importance of this strategy. Recently, Feng et al. showed that DNA detection could be improved using streptavidin coated AuNPs (Feng et al., 2013). Another interesting strategy was reported by Oh et al. where by modulating the FRET efficiency between QDs and AuNPs they were able to detect molecules which inhibited the interaction between streptavidin and biotin (Figure 14) (Oh et al., 2005). By capping the AuNPs with polyamidoamine dendrimers, the biotinylation was possible using sulfo-NHS-biotin.

A similar strategy was employed in the biotinylation of MNPs. To achieve a MNPs-PEG-biotin conjugate the NPs were incubated with a phospholipid-PEG-biotin construct. By coupling DC_14:0_ PE (dimyristoylphosphatidylethanolamine) to further activate α-biotinylamido-ω-N-hydroxy-succinimidcarbonyl-PEG, the authors could produce MNPs covered with PEG-biotin. The functionalization was confirmed when binding streptavidin alkaline phosphatase the complexes became highly aggregated (Hodenius et al., 2012).

The strong association between avidin and biotin has made this system a reference for the development and troubleshooting of NPs-based biosensors. It is also a crutch for conjugation of other biomolecules onto the surface of NPs. However, it is important to note that avidin is a glycoprotein with a high isoelectric point (~10). This could cause the unspecific binding of other compounds present in complex biological samples. To overcome this problem, it is preferred the use of streptavidin. As it is purified from a bacteria (*Streptomyces avidinii*) is not a glycoprotein and has much lower isoelectric point (around 5–6). Besides, the tetrameric nature of each (strept) avidin molecule becomes a problem when control of the Ab stoichiometry is needed. To overtake this problem, it is possible to use recombinant monomerics forms of these proteins but taking into account that the affinity for biotin would be much lower (around 10^−7^ M) (Wu et al., 2009b).

## Conclusions and future perspectives

In the near future, it is expected that the design of nanosystems will revolutionize the medical healthcare field by their application in the development of ultrasensitive and multiplexed diagnostic systems, targeted and remotely controlled drug delivery systems for treatment of diseases, *in vivo* imaging, tissue/organ regeneration and gene therapy solutions. The last three decades have been an exciting period in the synthesis of inorganic nanoparticles with interesting intrinsic properties for their use in such applications. Indeed, many of these synthetic processes have not only demonstrated proof-of-concept feasibility but progressed to full-scale commercial production. However, optimization of appropriate size scale and batch-to-batch reproducible synthetic procedures of NPs with unique optical or magnetic properties is not sufficient to ensure biomedical application. For this, functionalization of the NPs with biomolecules is crucial in order to impart biological recognition and interaction skills.

Selecting the most adequate biofunctionalization strategy is no mean feat, since no universal methodologies exist to cover the wide variety of inorganic nanoparticles and biomolecules available for this purpose. A functionalization protocol that works well for one type of NP may not work for another, since they could be very different in terms of size, charge, surface area, colloidal stability, density and type of reactive groups, etc. Furthermore, biomolecules vary significantly in terms of size, chemical composition, 3D complexity and location of its biological active site. As discussed along this review, in absence of standard functionalization protocols, each particular case (nanoparticle + biomolecule) requires optimization. Thus, in addition to the development of “smart” multifunctionalization strategies, it is vital to focus on the synthesis of “smart” nanoparticles over the next decade. These NPs should be able to deliver a therapeutic agent based on environmental causes or remote stimulus and with the capability to temporarily adapt their size, shape, surface chemistry, wettability and adhesive properties to surrounding environments. These long-term goals would allow an overall impact on the medical field with significant advances in patient screening, monitoring, diagnosis, staging, and treatment.

### Conflict of interest statement

The authors declare that the research was conducted in the absence of any commercial or financial relationships that could be construed as a potential conflict of interest.
